# Four-week supplementation with a blackcurrant formulation enhances cognitive performance, reduces mental fatigue and modulates peripheral monoamine metabolism in older adults: a randomized, double-blind, placebo-controlled, crossover study

**DOI:** 10.3389/fphar.2026.1896655

**Published:** 2026-09-30

**Authors:** Dominic Lomiwes, Catrin S. Günther, Alexander P. Kanon, Kim Lo, Andrew Scholey, Nayer Ngametua, Tania M. Trower, Edward G. Walker, Dwayne A. Jensen, Janine M. Cooney

**Affiliations:** 1 Health and Consumer Sciences Group, New Zealand Institute for Bioeconomy Science Limited, Palmerston North Campus, Palmerston North, New Zealand; 2 Biological Chemistry and Bioactives Group, New Zealand Institute for Bioeconomy Science Limited, Ruakura Campus, Hamilton, New Zealand; 3 Health and Consumer Sciences Group, New Zealand Institute for Bioeconomy Science Limited, Mount Albert Campus, Auckland, New Zealand; 4 School of Psychology, University of Northumbria, Newcastle upon Tyne, United Kingdom

**Keywords:** blackcurrant, cognitive function, l-Theanine, monoamine oxidase, mood, neurotransmitters, older adults

## Abstract

**Introduction:**

Age-related cognitive decline is an increasing concern in older adults. While blackcurrants (*Ribes nigrum*) have shown promise in improving cognitive outcomes in younger adults, their efficacy in supporting cognitive function in older adults is largely unexplored.

**Methods:**

In this randomized, placebo-controlled, double-blinded crossover study of healthy older adults (62 ± 1 years), 26 of the 30 participants enrolled completed the study per protocol. Participants consumed either a placebo or a flavonoid-rich blackcurrant formula (BCF, 7.8 mg anthocyanins/kg body weight freeze-dried blackcurrant, 200 mg L-theanine, and 100 mg pine bark extract) in a beverage format daily for 4 weeks, with interventions separated by a two-week washout period. For each intervention, cognitive and mood outcomes were assessed following a single dose (acute) and after 4 weeks’ supplementation. Platelet MAO-B activity, circulating neurotransmitter concentrations, and anthocyanin, L-theanine and sarmentosin bioavailability were measured from venous blood samples drawn at baseline, 1 h, 2.4 h and 4 wk after intervention supplementation.

**Results:**

A significant reduction in Rapid Visual Information Processing (RVIP) false alarms (*p* = 0.022) and a strong trend in reduced mental fatigue (*p* = 0.046, *q* = 0.067) were measured following 4 weeks of BCF supplementation. Reduction in biomarkers of MAO activity were measured following acute supplementation with BCF, but not placebo, with significant treatment × time interactions (*q* < 0.01) for platelet MAO-B activity, plasma 5-hydroxyindoleacetic acid and 3,5-dihydroxyphenylglycine concentrations. These changes corresponded with significant reductions (*q* < 0.05) in circulating neurotransmitter metabolites xanthurenic acid, 3-methoxy-4-hydroxyphenylglycol and vanillylmandelic acid.

**Conclusion:**

Findings from this study indicate that 4 weeks of BCF supplementation sustained attention in healthy older adults, extending evidence for BCF-related cognitive benefits across both younger and older populations.

**Clinical Trial Registration:**

https://anzctr.org.au/, identifier: ACTRN12621001590853.

## 1 Introduction

Declining cognitive function is a growing global concern among ageing populations, affecting millions of older adults and substantially affecting their quality of life (Salari et al., 2025). Although the underlying causes for age-related cognitive deficits are complex and remain poorly understood, healthy lifestyle habits including regular physical activity, a balanced diet and cognitive exercises are gaining attention as potential strategies for maintaining cognitive function and mental resilience (Potashkin et al., 2025). Dietary interventions rich in natural bioactive compounds have also been a promising research focus owing to established neuroprotective properties. For example, a recent meta-analysis suggests that supplementation with anthocyanin-rich foods may improve performance across multiple cognitive domains including attention, processing speed and memory (Micek et al., 2025).

Blackcurrants (*Ribes nigrum*) are rich in both anthocyanins and the γ-nitrile glycoside sarmentosin (Lomiwes et al., 2024), and therefore a promising candidate for dietary intervention strategies targeting cognitive performance and mood in older adults. Randomised, placebo-controlled studies have demonstrated that a single dose of blackcurrants (BC) can improve reaction times during cognitive fatigue (Watson et al., 2015), and maintain a positive mood and lower perceived exertion during moderate exercise (Lomiwes et al., 2019). The effect of BC consumption on acute modulation of electroencephalogram α- and β-waves supports an anxiolytic effect while concurrently improving attention (Watson et al., 2019). These cognitive and mood benefits from BC consumption have been attributed to their efficacy in maintaining neurotransmitter balance by transiently peripheral monoamine oxidase (MAO) activity. This is reflected in peripheral reductions in peripheral MAO-B activity, 3,5-dihydroxyphenylglycine (DHPG) (reflective of MAO-A inhibition), and modulation plasma prolactin concentration, all suggesting enhanced central dopamine (DA) signalling (Watson et al., 2015).

Other plant-derived supplements such as L-theanine and pine bark extract also support cognitive function through distinct mechanisms. L-theanine has been reported to promote relaxation without drowsiness, with both clinical and pre-clinical research demonstrating its efficacy in improving mood through the modulation of central monoaminergic and glutamatergic neurotransmitter signalling (Nathan et al., 2006; White et al., 2016; Hidese et al., 2019). Supplementation with proanthocyanidin-rich pine bark extract has been associated with improvements in memory, executive function and mental clarity. These effects may be mediated by its antioxidant properties and ability to enhance endothelial function and cerebral blood flow through nitric oxide regulation (Simpson et al., 2019).

Given their distinct biological activities, combining BC, L-theanine and pine bark extract may support mood and cognitive function through multiple complementary mechanisms. While BC influence monoamine neurotransmitter metabolism through MAO inhibition, L-theanine modulates glutamatergic and monoaminergic signalling, and pine bark extract may improve cerebral blood flow while reducing oxidative stress. By collectively acting on neurochemical, vascular and antioxidant pathways which have been associated with cognitive performance and psychological wellbeing, ingredients may provide additive or synergistic benefits beyond those achieved by any single ingredient alone.

Ārepa®, a nootropic formulation containing Neuroberry® BC, L-theanine and pine bark extract (Enzogenol®), has been shown to enhance cognitive performance in several cohorts. Short-term supplementation improved attention and working memory during acute ozone exposure (Morton et al., 2024b) and post-training recovery in athletes (Gibson et al., 2020). Importantly, these improvements were associated with augmented circulating brain-derived neurotrophic factor (BDNF) (Morton et al., 2024a), indicating a potential role of the active intervention in maintaining neuronal plasticity and function. Four-week supplementation with this formulation was also reported to enhance working memory while reducing mood domains associated with anxiety and anger in healthy adult women during a multitasking challenge (Gillies et al., 2024).

To date most studies investigating the cognitive and mood-enhancing effects of Ārepa’s BC, L-theanine and pine bark extract formulation (BCF) is largely limited to younger adult populations (mean age 29.8–43.8 years), with only one study investigating the effect of Ārepa’s BC powder on cognitive function in older adults (mean age 69 years) (Do Rosario et al., 2026). To address this gap, we have previously reported that 4 weeks of daily supplementation with BCF improved sleep quality in adults aged 55 years and older (Lomiwes et al., 2025b), indicating potential neurobehavioural benefits in older adults, although associations with neurotransmitter chemistry remained to be investigated. Building on these findings, the present study reports primary outcomes from the same cohort, investigating the effects of acute and four-week daily consumption of Ārepa’s BCF on cognitive performance and mood in older adults during a cognitive battery in a double-blinded, placebo-controlled study design. To elucidate potential pathways that may mediate any observed functional effects, platelet MAO-B activity and circulating neurotransmitters were also quantified and data integrated with previously reported outcomes on sleep quality (Lomiwes, Kanon, Walker, et al., 2025).

## 2 Materials and methods

### 2.1 Study design

This study utilized a randomized, double-blinded, placebo-controlled crossover intervention design and was conducted from September 2022 to May 2023 at New Zealand’s Bioeconomy Science Institute (BSI), Auckland. The study protocol and procedures were reviewed and approved by New Zealand’s Northern B Health and Disability Ethics Committee (2021 EXP 11576). The study was conducted in accordance with the Declaration of Helsinki guidelines and prospectively registered with the Australia New Zealand Clinical Trials Registry (ACTRN12621001590853).

### 2.2 Participants

Healthy males and females aged 55 years and older were recruited to this study. Eligible participants self-reported habitual low consumption of polyphenol-rich foods and were non-smokers and non-vapers. Participants also completed the Mini-Mental State Examination (MMSE) (Folstein et al., 1975) to assess cognitive impairment, and those with a score greater than 20 were eligible to participate. Other inclusion and exclusion criteria to determine whether prospective participants were eligible to take part in this study have been previously detailed by Lomiwes et al. (2025b).

An *a priori* power analysis was conducted using Minitab® (version 22.2.2) to determine the required sample size. The analysis was based on the Digit Vigilance and Rapid Visual Information Processing (RVIP) results reported by (Watson et al., 2015) examining the effects of a single dose of blackcurrant juice in maintaining attention during repeated cognitive tasks in healthy adults aged 18–34 years. The analysis indicated that sample sizes of 22 and 15 participants would be sufficient to detect a between-treatment difference in Digit Vigilance and RVIP, respectively, at an alpha level of 0.05 with 80% statistical power. To allow for an anticipated dropout rate of approximately 20% and potential non-compliance, 36 participants were enrolled in this study.

Fifty-four individuals expressed interest in participating in this study. Thirty-six were eligible and provided signed consent prior to being enrolled, then randomly assigned to either the BCF group (n = 18) or the placebo group (n = 18) before entering the first study arm. After completing the first study arm, participants underwent a washout period of at least 2 weeks, during which they were asked to maintain habitually low consumption of polyphenol-rich foods. Following the washout period, participants began the second study arm, this time supplementing with the opposite intervention to their first study arm (Figure 1A). This study was conducted in accordance with the Consolidated Standards of Reporting of Trials (CONSORT) guidelines, as detailed in Figure 2 which was adapted and modified from Lomiwes et al. (2025b).

**FIGURE 1:**
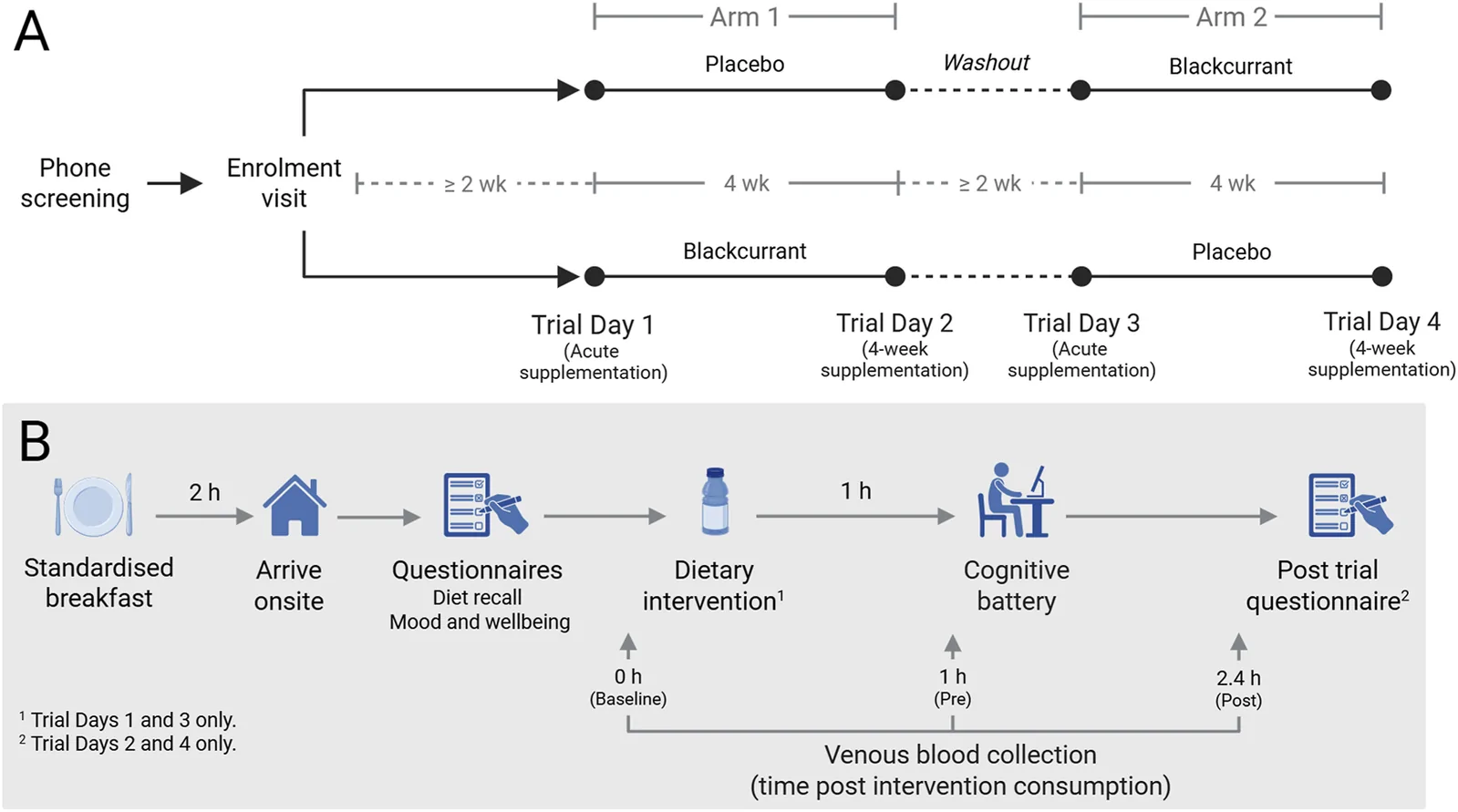
Study design overview showing trial timelines, treatment arms, and washout periods **(A)**, and the procedures and assessments conducted on each trial day **(B)**. Figure was created using BioRender.com.

**FIGURE 2:**
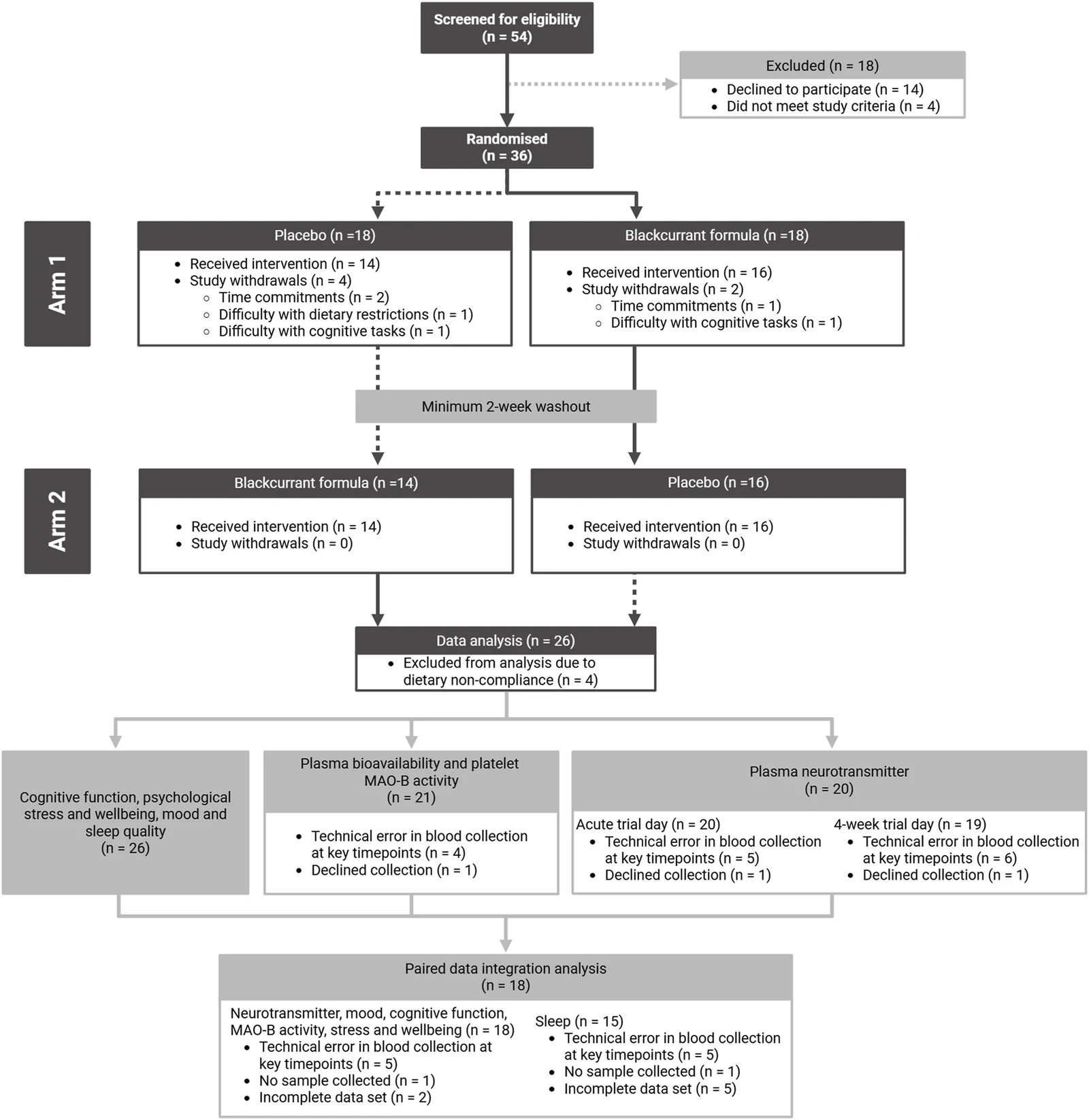
Consolidated standards of reporting trials (CONSORT) flow diagram detailing the progression of participants through the study.

### 2.3 Treatments

The study’s BCF and placebo interventions and participant supplementation regimen have been previously detailed in Lomiwes et al. (2025b). Briefly, randomized allocation of participants to either the BCF (Ārepa® Brain Powder) or placebo interventions was conducted by an individual who was independent from the study, ensuring both researchers and participants were blinded to the allocated interventions. Each dose of BCF contained 200 mg L-theanine (200 mg), 100 mg pine bark extract (Enzogenol®) and Neuroberry® BC powder corresponding to 7.8 mg anthocyanins/kg bodyweight (Table 1). This dose would also provide 3.2 mg of sarmentosin/kg of bodyweight based on previous compositional analysis of the study’s BC powder (Lomiwes et al., 2024). The placebo intervention was formulated to closely resemble the BCF intervention in taste, texture and appearance once reconstituted in water. Participants were instructed to reconstitute their allocated intervention in 250 mL of water by shaking for 30 s using a provided drink shaker, and to consume the beverage immediately after preparation. The BCF was supplied by Ārepa IP Ltd (Auckland, New Zealand), and the placebo was prepared by Sensient Technologies (Auckland, New Zealand).

**TABLE 1:** Amounts of anthocyanin, sarmentosin, L-theanine and pine bark extract in a single dose of Blackcurrant formula or Placebo. Data are means ± SEM.

Intervention constituent	Blackcurrant formula		Placebo	
​	Males (n = 8)	Females (n = 18)	Males (n = 8)	Females (n = 18)
Anthocyanins				
Normalised (mg/kg bodyweight)	7.8	7.8	0	0
Total per dose (mg)	645 ± 31	607 ± 33	-	-
Range per dose (mg)	543–819	406–952	-	-
Sarmentosin				
Normalised (mg/kg bodyweight)	0.32	0.32	0	0
Total per dose (mg)	26 ± 1.3	24 ± 1.3	-	-
Range per dose (mg)	22–33	16–38	-	-
L-theanine				
Normalised (mg/kg bodyweight)	2.5 ± 0.1	2.7 ± 0.1	0	0
Total per dose (mg)	200	200	-	-
Range per dose (mg/kg bodyweight)	1.9–2.9	1.6–3.8	-	-
Pine bark extract				
Normalised (mg/kg bodyweight)	1.2 ± 0.1	1.4 ± 0.1	0	0
Total per dose (mg)	100	100	-	-
Range per dose (mg/kg bodyweight)	1.0–1.4	0.8–1.9	-	-

### 2.4 Study procedures

Eligible participants attended a familiarization visit at least 1 month prior to study commencement, during which they were introduced to all cognitive tasks and study questionnaires administered at each trial visit (Figure 1A). Participants completed a practice trial day (enrolment visit) at least 2 weeks before entering the first study arm, to assess their ability to complete the cognitive battery, comply with trial day procedures, and minimize learning effects that could confound cognitive outcomes. The enrolment visit was identical in logistics and procedures to all subsequent trial days, other than treatment administration.

The trial structure was designed to evaluate the effects of acute and four-week daily supplementation with BCF versus placebo on cognitive performance during a cognitively fatiguing battery. Psychological wellbeing, mood, and sleep quality were assessed as secondary outcomes using validated questionnaires as previously reported (Lomiwes et al., 2025b). Participants were instructed to abstain from anthocyanin-rich foods and caffeine 24 h prior to each trial day. On the morning of each trial day, participants consumed either a standardized meal bar (2900 kJ; One Square Meal® almond flavour, Cookie Time Ltd, Christchurch, New Zealand) or a low-polyphenol breakfast approved by the research team at least two hours before arrival at the BSI clinical trial facility.

Upon arrival, participants confirmed compliance with all pre-trial requirements, completed validated questionnaires assessing psychological wellbeing, mood, and sleep quality (Lomiwes et al., 2025b), and provided a venous blood sample (baseline). On acute supplementation trial days (Trial Days 1 and 3), participants then consumed their allocated intervention, followed by collection of a second blood sample 1 h post-consumption. Participants subsequently completed a series of computer- and paper-based cognitive tasks designed to induce cognitive fatigue and stress, after which a final venous blood sample was collected (2.4 h post-consumption). At the end of each trial arm (Trial Days 2 and 4), participants completed a questionnaire with a number of close-ended questions, documenting any positive, negative, or unusual physical or mental health changes that they attributed to the study interventions (Figure 1B). Further details and findings from this study’s validated questionnaires and participants’ subjective experiences have been reported previously (Lomiwes et al., 2025b).

After completing the first dietary intervention arm, participants underwent a washout period of at least 2 weeks before entering the second study arm. This followed identical procedures as the first study arm, including daily supplementation over the four-week intervention period, except that participants received the alternate dietary intervention from the first study arm.

### 2.5 Cognitive and mood measures

All computer-based cognitive task and mood scales were administered using the Computerised Mental Performance Assessment System (COMPASS, Northumbria University, Newcastle Upon Tyne, UK). Cognitive performance across six domains of attention, working memory, executive function, episodic memory and processing speed was evaluated using a number of pre-selected tasks that formed the study’s test battery. Tasks were administered in a fixed order as follows: word presentation, digit vigilance, arrow flanker, Serial Sevens, symbol digit substitution, rapid visual information processing (RVIP) and delayed word recall. The cognitive battery was repeated three times in immediate succession, with mood scales completed before each repetition and following completion of the final test battery.

#### 2.5.1 Delayed word recall

The delayed word recall task assessed episodic memory.

*Word presentation:* Prior to each repetition of the cognitive test battery, participants were presented with a sequence of 15 random words over a 60-s period.

*Word recall:* After the final task in each test battery repetition (i.e. the RVIP task), participants were instructed to write down as many words as they could recall from the list presented at the beginning of that battery. Participants were given 60 s to complete this task.

#### 2.5.2 Digit vigilance

The digit vigilance task assessed sustained attention and vigilance with minimal demands on selective attention and working memory. Participants viewed a continuous sequence of single digits presented on the left side of the computer monitor and were instructed to press the spacebar whenever the displayed digit matched a predefined target digit displayed on the right side of the screen. The task lasted 2 min and included 30 target matches. Outcome measures comprised response accuracy (%) and reaction time (ms) for both correct and incorrect responses.

#### 2.5.3 Arrow flanker

The arrow flanker task measures selective attention. In this test, five symbols appear horizontally across the computer monitor, with the centre symbol an arrow either pointing to left or right. The flanking symbols are either arrows pointing in the same direction (congruent), the opposite direction (incongruent) or appear as squares (neutral). Participants are instructed to indicate which direction the centre arrow is pointing by pressing on the corresponding symbols key on the computer keyboard or pressing on the spacebar if the arrow is flanked by squares. Sixty stimuli were presented and task outcomes were accuracy (overall, congruent and incongruent stimuli) and reaction time (ms) for correct and incorrect responses (overall, congruent and incongruent stimuli).

#### 2.5.4 Serial Sevens

This task assesses working memory and executive function. It starts with a random number between 800 and 999 presented in the computer monitor. Participants are required to subtract seven from the given number as quickly and as accurately as possible and enter their response using the numbers key on the computer keyboard. They are instructed to continue to subtract from the resultant numbers as quickly and as accurately as they can for two minutes. Task outcomes include total number of responses, the number of correct and incorrect responses and participant’s accuracy during the task.

#### 2.5.5 Symbol digit substitution

The digit symbol substitution test (DSST) is a subset of the intelligence test introduced by Wechsler (1958). In this paper-and-pen coding task, the digits 1 to 9 were matched with nine different symbols presented at the top of the task sheet. Below the symbol-digit code are five rows, each consisting of 25 numbers. Starting from the first row, participants are instructed to write the corresponding symbol below each number according to the key presented at top of the worksheet as quickly as they can.

In this study, we used the symbol digit substitution test (SDST), a reversed version of the DSST. In this version, participants are presented with symbols and instructed to write the corresponding numbers beneath each symbol according to the key at the top of the test sheet. This approach was chosen to avoid the motor planning required for drawing complex symbols. Task outcomes included the number of responses completed within 90 s and accuracy of responses (as a percentage of correct versus incorrect responses).

#### 2.5.6 Rapid visual information processing

The RVIP task measures attention and working memory. Participants were presented with a quick succession of single digits on the monitor at a rate of 100 numbers per minute. Participants are required to respond by pressing on the spacebar of the computer keyboard as quickly as they can when they see three consecutive even or odd numbers. The task lasts for 5 min. Task outcomes include accuracy (percentage of correct responses detected), reaction time to correct responses and number of incorrect responses.

#### 2.5.7 Bond-Lader and visual analogue scales

Collection and collation of Bond–Lader Mood Scales data were conducted as previously described by Lomiwes et al. (2025b).

Participants were asked to subjectively rate how stressed, anxious and mentally fatigued they felt at the start then after each repetition of the cognitive battery using individual visual analogue scales. Each scale was 100 mm and were anchored by descriptors “not at all” and “extremely” on the left and right ends of each scale, respectively.

#### 2.5.8 Sleep quality

Participant sleep quality was assessed using the Pittsburgh Sleep Quality Index (Buysse et al., 1989). Administration and results from this questionnaire have been previously described by Lomiwes et al. (2025b).

### 2.6 Analysis of biological samples

At each venous blood collection timepoint, blood samples were drawn into vacutainer tubes containing anticoagulants lithium heparin (10 mL) and ethylenediaminetetraacetic acid (EDTA; 10 mL). Separation of plasma and isolation of platelets from lithium heparin and EDTA vacutainer tubes, respectively, were conducted using procedures previously described (Lomiwes et al., 2024).

#### 2.6.1 Quantitation of plasma anthocyanin, sarmentosin and L-theanine bioavailability

Analysis of plasma aliquots from blood samples collected in lithium heparin-coated vacutainers for anthocyanin, sarmentosin and L-theanine bioavailability were conducted as detailed in previous publications (Lomiwes et al., 2024, 2025b).

#### 2.6.2 Quantitation of neurotransmitters in plasma

Plasma from lithium heparin blood samples were analysed for neurotransmitters from the tyrosine (TYR) and tryptophan (TRP) pathways, along with their precursors and catabolites, using two separate workflows as detailed in Lomiwes et al. (2024).

#### 2.6.3 Platelet MAO-B activity

Platelet MAO-B activity was measured using the Amplex® Red Monoamine Oxidase kit (Cat. No. A12214, ThermoFisher Scientific, Auckland, New Zealand), according to procedures described by Watson et al. (2015). The protein concentration of the platelet samples was determined using the Pierce™ Bicinchoninic Acid protein assay kit (Cat. No. 23255, ThermoFisher Scientific, Auckland, New Zealand).

### 2.7 Inhibition of MAO-B by blackcurrant formulation and its constituents

The efficacy of BCF and individual constituents of the BCF formulation (i.e. BC powder, L-theanine and Enzogenol®) in inhibiting MAO-B enzyme activity was measured using the Promega MAO-Glo™ assay (Cat. No. V1401. *In Vitro* Technologies, Auckland, New Zealand) according to the manufacturer’s instructions. Because of the sample colour of BCF and BC powder interfering with luminescence readings from the assay, testing of enzyme inhibition was conducted at concentrations that did not interfere with assay responses.

### 2.8 Statistical analysis

Statistical analyses of all cognitive and mood outcomes, bioavailability and MAO enzyme activities were conducted using Minitab®. Analyses of data from acute and 4-wk trial days were conducted using linear mixed-effects models. The number of participants included in each analysis is reported in Figure 2. For cognitive tests, fixed effects were set for timepoint (acute and 4-wk), treatment (placebo and BCF), and its interaction, and random effect for participant. For mood scores, fixed effects were set for timepoint (acute and 4-wk), treatment (placebo and BCF), repetition (pre- and post-cognitive test), and its interactions, and random effect for participant. For both bioavailability and MAO activity data, fixed effects were set for timepoint (baseline, 1 h, 2.4 h and 4 wk), treatment (placebo and BCF), and its interaction, and random effect for participant. To assess potential carryover effects during the wash-out period, acute supplementation data were additionally analysed using fixed effects for treatment order and treatment order × timepoint interaction. Assumptions for normality and symmetry of mixed effects models were confirmed using residual Q-Q and histogram plots, and residuals versus fitted value plots were examined to confirm homoscedasticity. *Post hoc* Tukey analysis was conducted when significance for main effects or interactions was observed. Given the large number of secondary cognitive, mood, and biochemical outcomes evaluated, *p*-values for fixed effects and their interactions were adjusted for multiple testing using a False Discovery Rate (FDR) approach. Adjusted *q*-values were calculated using the Benjamini, Krieger, and Yekutieli two-stage step-up procedure in GraphPad Prism (version 10.6.1), unless otherwise stated, and reported alongside *p*-values for secondary cognitive and mood outcomes.

Unless otherwise stated, visualization and data analysis of neurotransmitters and data integration was performed in R version 4.4.3. To enable a paired analysis design for the comparison of acute and 4-week effects of participants, the datasets were curated to include only participants (n = 18) for which both placebo and BCF-intervention data were available from both study arms. A small amount of random missing data (<2% of the data set) from these participants, were imputed using multivariate imputation by chained equations as implemented in mice (Buuren and Groothuis-Oudshoorn, 2011). To compare changes in neurotransmitter metabolites following acute and 4-wk supplementation, data for each timepoint (baseline, pre- and post-cognitive testing) were expressed as proportional changes from placebo and visualized as blobbograms using the ggforestplot package (Scheinin et al., 2025). Parametric 2-way repeated measures ANOVA was performed with ‘treatment’, ‘study-arm’ and their interactions as explanatory variables. This was followed by pairwise comparisons using paired t-test and false discovery rate adjustment for multiple comparisons at α = 0.05. Normality was confirmed using the Shapiro-Wilk test. For comparative analysis of neurotransmitter metabolites after acute supplementation and their relationship with MAO-B inhibition, data from the acute trial days were normalised to proportional changes from baseline. MAO-B inhibition was expressed as the inverse of MAO-B activity. Heatmaps were generated using ‘gplots’ (Warnes et al., 2005) and bargraphs using ‘ggplot2’ (Wickham, 2016). Graphics were manually curated to include statistical outputs from paired t-tests and Pearson correlation analysis, respectively. Pearsons-correlation analyses of neurotransmitter and previously-reported 4-wk sleep quality outcomes (Lomiwes et al., 2025b), normalized to pre-supplementation (baseline) values, was conducted using the ‘psych’ package (Revelle, 2026) and visualized as chord-plots using “circlize” (Gu et al., 2014) in combination with “ComplexHeatmap” (Gu et al., 2016). To assess potential attrition bias resulting from the substantial number of participants excluded from the paired data integration analysis, two-sample t-tests (Minitab®) were performed to compare anthropometric measures, primary cognitive outcomes, MAO-B inhibition biomarkers, and selected mood and sleep measures between participants included in and excluded from the analysis.

## 3 Results

Of the 36 participants enrolled, 30 participants (22 females and eight males) completed the study (Figure 2). However, data from four participants were excluded from the study because of non-compliance to dietary restrictions, as revealed by the presence of L-theanine and/or anthocyanins in their blood. Of the compliant participants, who completed the study, datasets for cognitive function outcomes, stress and wellbeing, and sleep measures were available for 26 individuals. Owing to insufficient sample volume, technical issues in blood collection, and a participant declining to provide a blood sample, available paired datasets were further reduced for neurotransmitters, plasma bioavailability and MAO-B activity measures. Consequently, MAO-B analyses included samples from 21 participants, while plasma neurotransmitter data were available from 20 (acute trial) and 19 (4-wk trial) participants. Across the entire trial, paired data were available from 18 participants and included in integrative analyses of neurotransmitters with MAO-B activity, cognitive, mood and wellbeing outcomes (Figure 2).

While the study cohort was predominantly female, treatment allocation was sex-balanced so that exactly an equal number of males (n = 4) and an equal number of females (n = 11) were initially assigned to the placebo and BCF interventions. No significant differences in age or anthropometric measures were identified for males and females (Table 2). The study’s dietary interventions were well tolerated during both acute trial days and over the 4-week supplementation period with no adverse events reported by study participants (Lomiwes et al., 2025b). The most unfavourable response given by a small number of participants was a dislike of the intervention’s taste, in both the BCF (n = 2) and placebo (n = 1) groups.

**TABLE 2:** Characteristics of participants who completed the study. Data are means ± SEM, with data range in brackets.

Participant characteristic	Placebo → BCF sequence		BCF → Placebo sequence	
	Male (n = 4)	Female (n = 9)	Male (n = 4)	Female (n = 9)
Age (years)	66 ± 3.7 (57–76)	60 ± 1.4 (55–68)	59 ± 2.0 (56–66)	64 ± 1.9 (59–74)
Height (cm)	175 ± 2.3 (170–182)	167 ± 1.9 (160–177)	173 ± 2.3 (165–177)	161 ± 1.4 (152–167)
Weight (kg)	84 ± 6.0 (76–105)	79 ± 20 (52–122)	81 ± 5.3 (70–96)	77 ± 5.2 (52–100)
BMI (kg/m^2^)	27 ± 1.2 (26–32)	29 ± 2.6 (21–47)	27 ± 1.5 (24–32)	29 ± 1.8 (22–40)
MMSE scores	29 ± 0.4 (28–30)	29 ± 0.6 (24–30)	30 ± 0.0	30 ± 0.1 (29–30)

MMSE, mini mental state examination.

### 3.1 Four-week BCF supplementation improved attention while reducing and mental fatigue

Of the cognitive tests conducted in this study, only the RVIP task showed significant benefit from BCF supplementation (Supplementary Table S1). A significant treatment × interaction effect was observed (*p* = 0.009) for false alarms during the RVIP test, with no main effects observed for time (*p* = 0.128) or treatment (*p* = 0.324). *Post hoc* analysis indicated that false alarms significantly decreased after 4 weeks of BCF supplementation compared with when participants consumed an acute dose (7.3 ± 1.3 vs. 5.5 ± 0.9, acute vs. 4 wk; *p* = 0.022). No such change was observed when participants consumed the placebo intervention (Figure 3A). When expressed as absolute change from acute supplementation, a significant treatment effect (*p* = 0.007) was measured, indicating the reduction in false alarms following 4 weeks of BCF, which was not observed in the placebo group (0.7 ± 0.7 vs. −2.3 ± 0.7, placebo vs. BCF).

**FIGURE 3:**
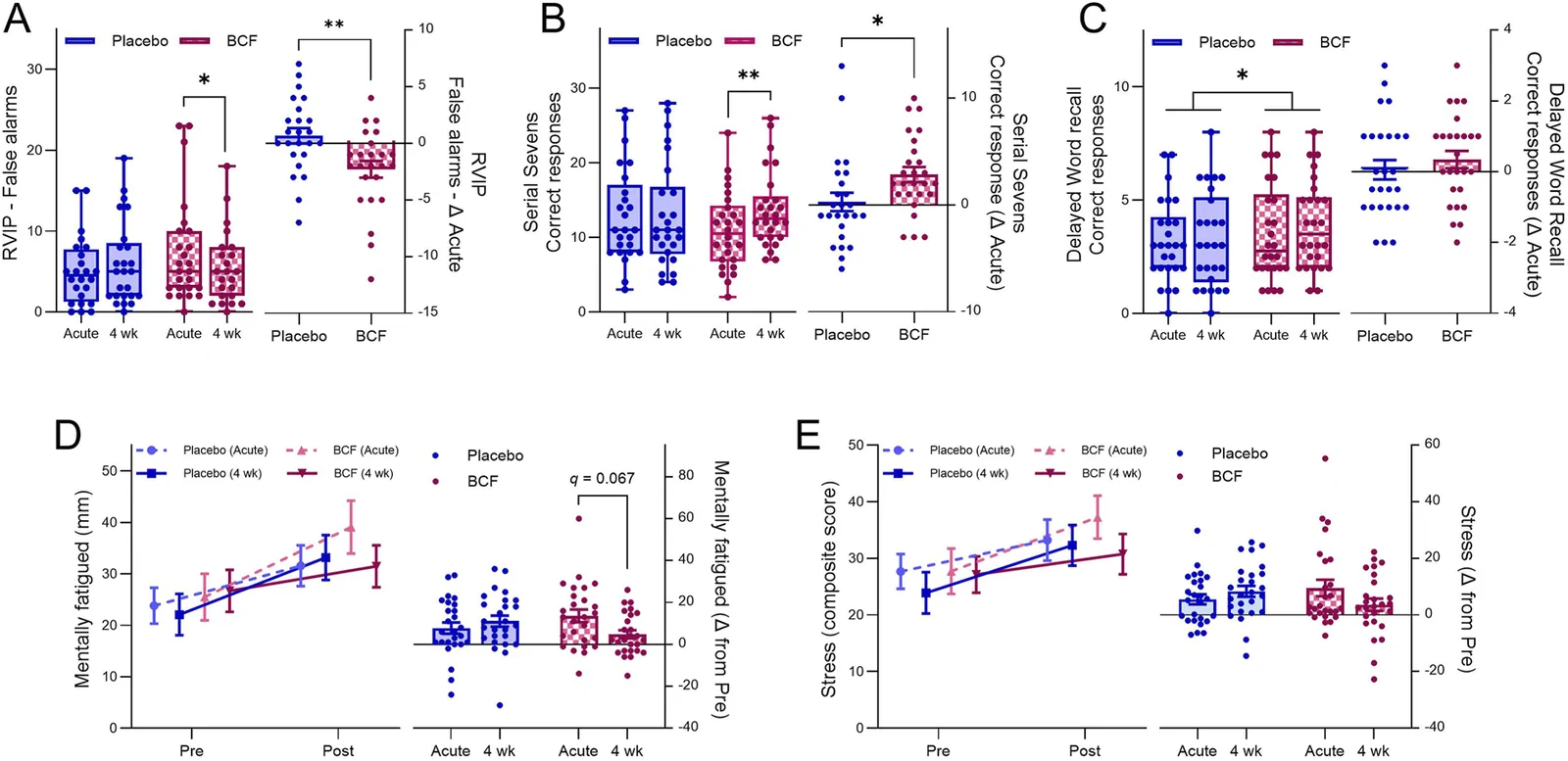
Cognitive outcomes following acute and 4 weeks of supplementation with the blackcurrant formulation (BCF) or placebo. Rapid Visual Information Processing false alarms **(A)**, Serial sevens correct responses **(B)**, and Delayed Word Recall correct responses **(C)** following acute and 4-week supplementation. Bond-Lader composite mental fatigue score **(D)** and stress score **(E)** were measured pre- and post-cognitive testing following acute and 4-week supplementation with the study interventions. Data are presented as means ± SEM. Asterisks indicate significant differences between timepoints or treatments (*p < 0.05; **p < 0.01).

For secondary cognitive outcomes, significant treatment × time interaction treatment was observed for correct responses during the Serial Sevens (Figure 3B, *p* = 0.026) task. The change in correct responses following 4 weeks of supplementation was also higher in the BCF group, compared with the placebo group (*p* = 0.025). Similarly, a significant main effect for treatment was observed for correct responses for the Delayed Word Recall task with higher correct responses in the BCF group than with the placebo group across both acute and four-week supplementary trial days (Figure 3C, *p* = 0.038). While the effects observed for these outcomes were no longer significant following FDR adjustment (*q* > 0.05; Supplementary Tables S1,S2), the trend in these responses suggest the potential for BCF supplementation in improving memory.

Absolute VAS ratings for mental fatigue increased significantly from pre-test values following a single repetition of the cognitive test battery in both treatment groups, irrespective of supplementation duration (*q* < 0.001; Supplementary Table S4). When mental fatigue scores were normalized to pre-test values, a near-significant treatment × time interaction was detected (*p* = 0.011, *q* = 0.067; Figure 3D) for this outcome; this corresponded with the mean reductions in mental fatigue experienced during the cognitive battery after 4 weeks of BCF supplementation (13.6 ± 3.0 vs. 4.8 ± 1.9 mm, acute vs. 4 wk). No such change was observed in the placebo condition.

Absolute Bond–Lader composite stress scores also increased significantly from pre-test values following a single repetition of the cognitive test battery (*q* < 0.001; Figure 3E). When stress scores during acute and four-week supplementation trial days were normalised to pre-test scores, a mean reduction in stress was noted in the BCF group (9.5 ± 2.9 vs. 3.6 ± 2.1, acute vs. 4 wk) but increased in the placebo group (5.5 ± 1.7 vs. 8.4 ± 1.9, acute vs. 4 wk). Despite these notable divergent trends, no significant treatment × time interaction was observed for this outcome (*p* = 0.042, *q* = 0.127) following FDR adjustment.

Treatment order or carryover effects did not significantly influence outcomes from these assessments, as no significant main effect were observed for treatment order or treatment order × trial day interactions for any cognitive or mood outcomes during the acute trial days (Supplementary Table S5, *q* > 0.05). that.

### 3.2 BCF modulates MAO activity and peripheral monoamine concentrations following an acute dose

Plasma bioavailability of BC anthocyanins, sarmentosin, and L-theanine were characterized following acute and four-week daily supplementation with the BCF and placebo interventions (Figures 4A–C). During the acute trial day, plasma concentrations of all three constituents changed dynamically, peaking at 1 h after BCF ingestion and declining thereafter. Plasma anthocyanin returned to concentrations comparable to those in the acute baseline by 2.4 h after BCF consumption (0.03 ± 0.01 vs. 9.7 ± 1.1 nM cyanidin 3-*O*-glucoside equivalents, baseline vs. 2.4 h; *q* = 0.060). In comparison, significant declines in sarmentosin (442 ± 40 vs. 323 ± 30 nM, *q* = 0.002) and L-theanine (32.1 ± 1.3 vs. 23.0 ± 1.4 µM; *q* < 0.001) were measured at 1 h and 2.4 h post BCF consumption, but these still remained measurably higher than acute baseline concentrations. Low, but detectable, concentrations of all three constituents were measured in plasma following 4 weeks of daily supplementation and collected at least 10 h after the final BCF dose. As expected, these constituents were undetectable, or present at negligible concentrations at all timepoints when participants received the placebo intervention (Supplementary Table S6).

**FIGURE 4:**
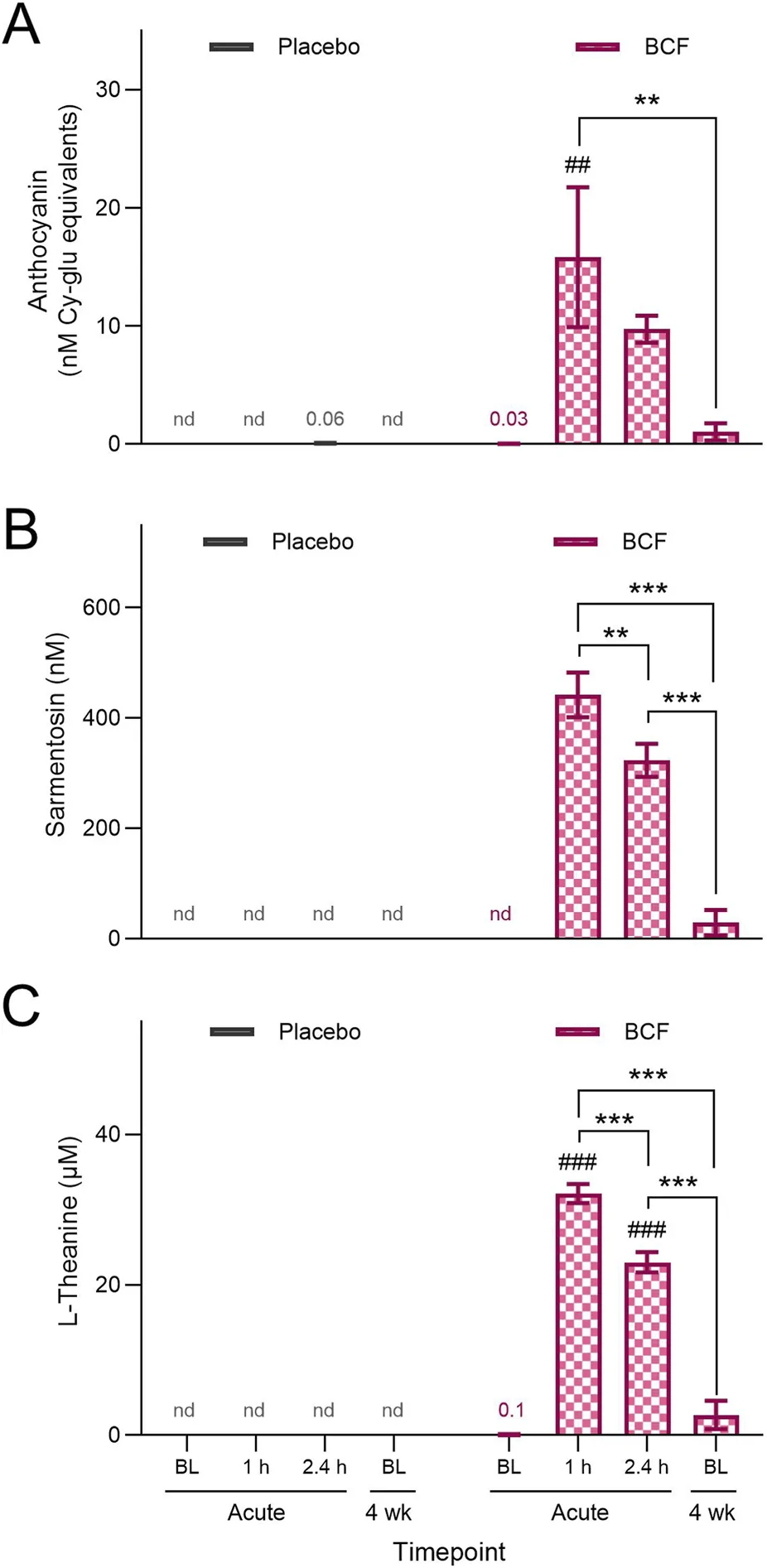
Plasma concentrations of total anthocyanins **(A)**, sarmentosin **(B)**, and L-theanine **(C)** at baseline (BL), 1 h and 2.4 h post supplementation, and after 4-week daily supplementation with the blackcurrant formulation (BCF) or placebo. Data are presented as means ± SEM. Values above bars are mean concentrations at the corresponding timepoint. Hash symbol indicates significant difference from baseline (##q < 0.01, ###q < 0.001). Asterisks indicate significant differences between timepoints (**q < 0.01; ***q < 0.001). nd, not detected.

Consistent with previous research, BCF supplementation significantly reduced platelet MAO-B enzyme activity compared with placebo, with significant main effects of treatment and time, as well as a treatment × time interaction (*q* < 0.001). Acute BCF supplementation significantly reduced MAO-B activity at 1 h and 2.4 h post-supplementation, timepoints corresponding to immediately pre- and post-cognitive testing, compared with baseline activity (19.3 ± 1.4, 1.5 ± 0.2 and 3.4 ± 0.5 nM H_2_O_2_/µg protein for baseline, 1 h, and 2.4 h, respectively; *q* < 0.001). MAO-B activity was significantly lower after 4 weeks of BCF supplementation than with the placebo (20.3 ± 1.4 vs. 16.2 ± 1.3 nM H_2_O_2_/µg protein, placebo vs. BCF; *q* = 0.003), indicating that four-week BCF supplementation sustained a small but significant reduction in platelet MAO-B activity (Supplementary Table S7).

Plasma 5-hydroxyindoleacetic acid (5-HIAA) and DHPG are catabolites generated primarily through MAO-A-mediated metabolism of serotonin (5-HT) and norepinephrine (NE), respectively. Consequently, circulating concentrations of these metabolites have been used as indirect biomarkers of MAO-A enzyme activity (Celada et al., 1990; Scheinin et al., 1991). A significant treatment × time interaction was observed for plasma 5-HIAA (*q* < 0.001; Figure 5B), with concentrations significantly reduced at 1 h and 2.4 h after consuming the first BCF dose compared with baseline (30.6 ± 2.0, 20.1 ± 1.7 and 22.3 ± 1.80 nM for baseline, 1 h, and 2.4 h, respectively). No such changes were observed in the placebo group (Supplementary Table S7).

**FIGURE 5:**
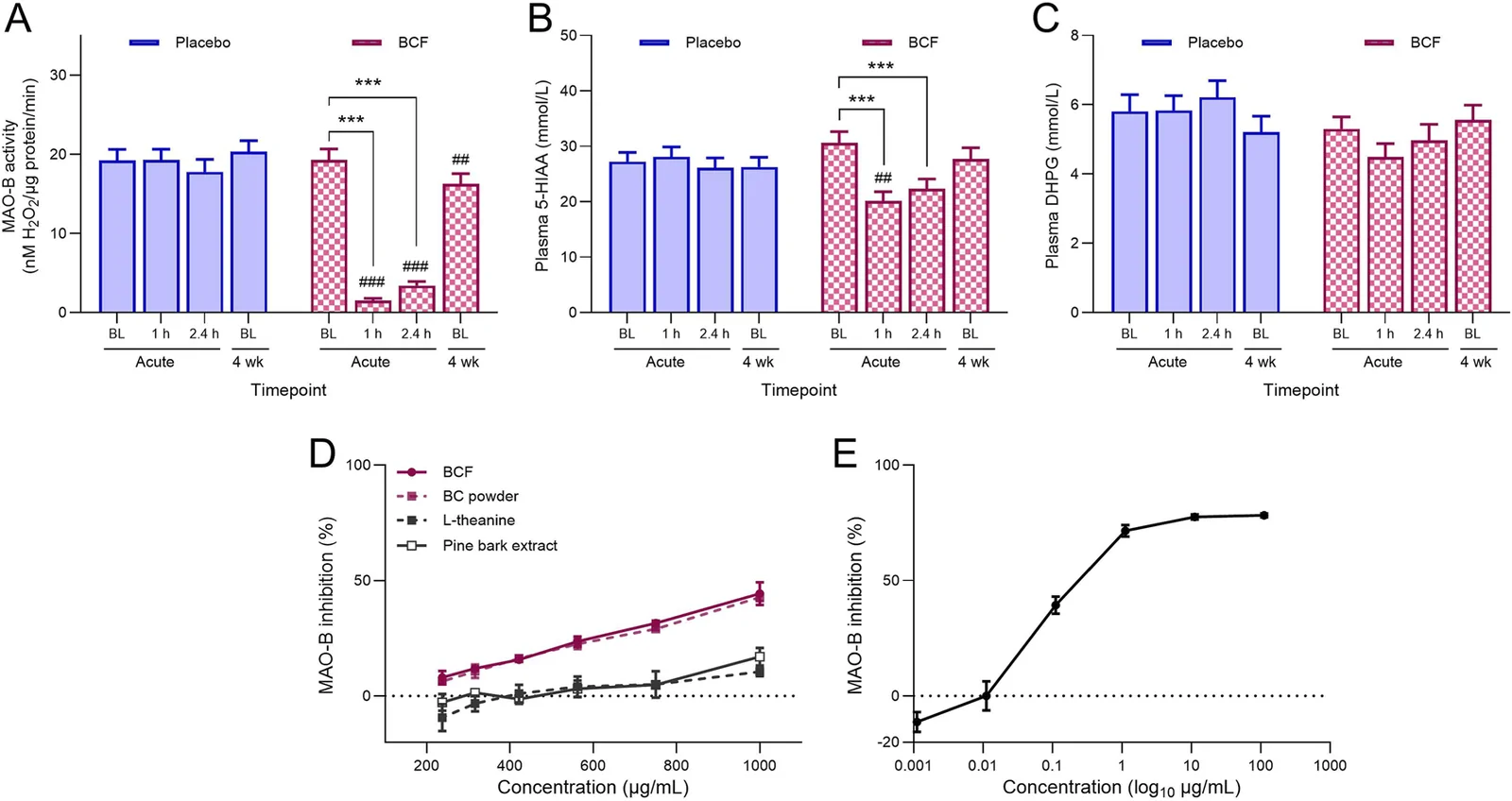
Platelet monoamine oxidase-B (MAO-B) activity **(A)** and plasma concentrations of 5-hydroxyindoleacetic acid (5-HIAA; **(B)** and 3,4-dihydroxyphenylethylene glycol (DHPG; **(C)** at baseline (BL), 1 h and 2.4 h post supplementation, and after 4 weeks of daily supplementation with blackcurrant formulation (BCF) or placebo. Dose-dependent effects of BCF and its individual constituents on MAO-B activity using the MAO-Glo™ assay **(D)**. MAO-B inhibition of individual constituents was tested at concentrations corresponding to that present in each BCF dose. Deprenyl hydrochloride is also shown as the positive control **(E)**. Data are means ± SEM. Asterisks indicate significant differences between timepoints (***q < 0.001). # indicates significant difference from the placebo group at the corresponding timepoint (##q < 0.01, (###q < 0.001).

Similarly, a significant main effect for treatment (*q* < 0.001) was observed for plasma DHPG, with mean concentrations across all timepoints being lower following BCF supplementation compared with the placebo (5.1 ± 0.2 vs. 5.8 ± 0.2 nM, respectively). in addition, a significant treatment × time interaction (*q* = 0.008) was detected. This is likely driven by mean reductions in DHPG concentrations following BCF supplementation, which remained largely unchanged in the placebo group.

The dose-dependent effect of each BCF constituent (i.e. BC powder, L-theanine and pine bark extract) was individually tested *in vitro* to determine the contribution of each constituent to BCF’s MAO-B inhibitory bioactivity. Results from the assay demonstrate that BC powder dose-dependently inhibited MAO-B similarly to the BCF. In comparison, equivalent concentrations of L-theanine and pine bark extract present in each BCF dose showed negligible effects on MAO-B activity (Figure 5D). When compared with deprenyl hydrochloride, an established MAO-B inhibitor, the highest BCF concentration and the equivalent BC powder concentrations achieved comparable MAO-B inhibition (approximately 40% inhibition) to that observed with 0.1 µg/mL deprenyl hydrochloride (Figure 5E). Taken together, these findings indicate that BC powder is the constituent with the largest contribution to the MAO-B efficacy of the BCF intervention.

No significant effects of treatment order or treatment order × trial day interaction were observed for any bioavailability or MAO inhibition outcome during the acute trial days (Supplementary Table S5; all q > 0.05), indicating an absence of treatment order or carryover effects on these study outcomes.

### 3.3 BCF modulates plasma neurotransmitter metabolite profiles

The effects of acute and four-week BCF consumption on plasma neurotransmitter concentrations of 15 TYR-derived and seven TRP metabolites were analysed using a repeated measures design.

To determine whether acute or four-week daily consumption of the BCF intervention modulated circulating neurotransmitter profiles, plasma concentrations were normalized within individuals to reflect proportional changes from placebo at corresponding time points on the acute and four-week trial days (Figure 6). Comparison of placebo-normalized concentrations showed significant reductions in the MAO products 5-HIAA (*q* = 0.04) and DHPG (*q* = 0.01) 1 h after BCF supplementation compared with baseline concentrations during the acute trial day (Figure 6). These findings further confirm the acute MAO-A inhibitory potential of the BCF intervention, and that this is not enhanced or sustained following 4 weeks of daily supplementation.

**FIGURE 6:**
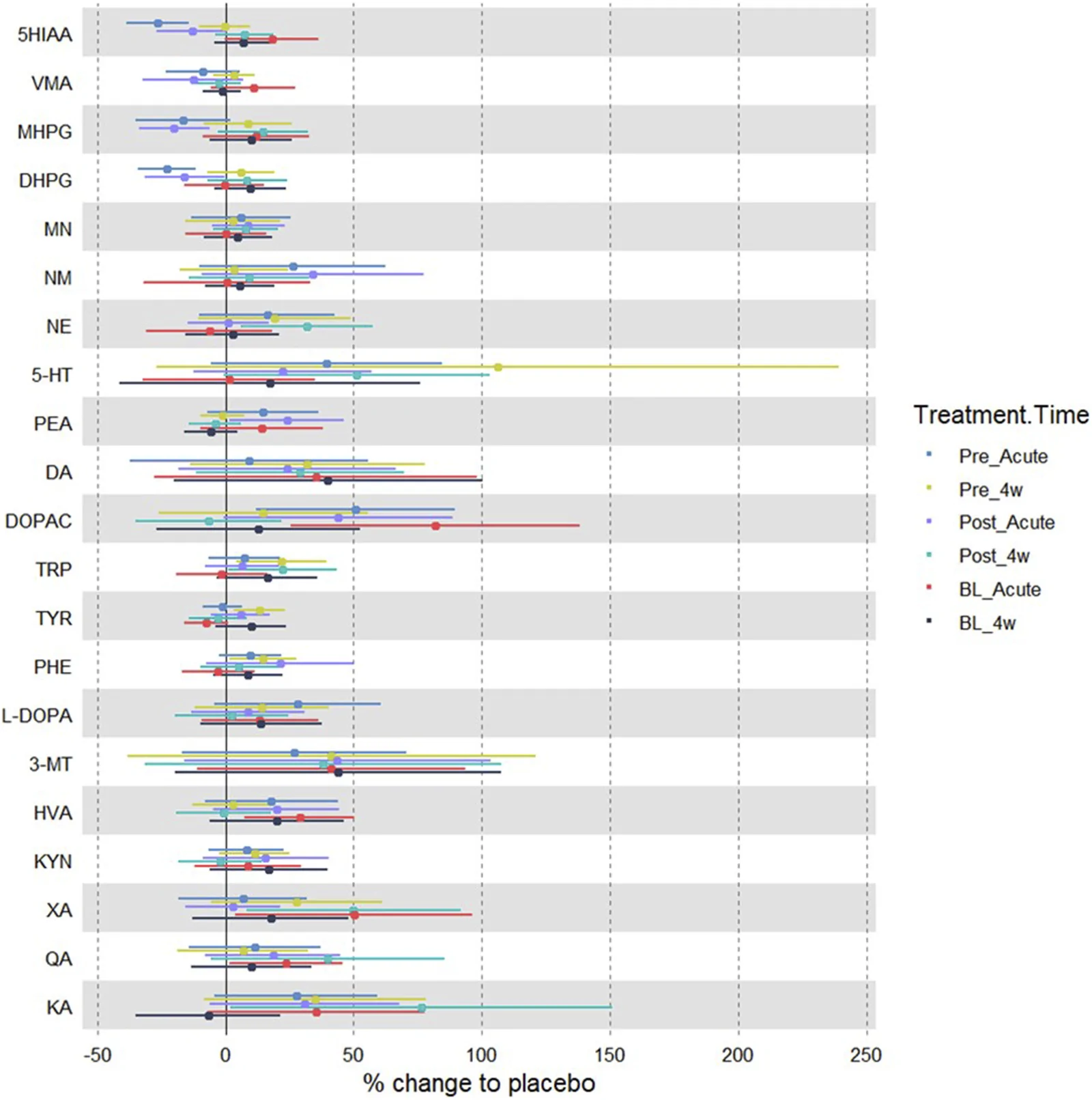
Blobbogram summarising mean percentage change from placebo (filled circles) and corresponding confidence intervals for targeted neurotransmitter metabolites following blackcurrant supplementation. BL, baseline; Pre, pre-cognitive testing; Post, post-cognitive testing; Acute, trial day when first dose administered; 4w, following daily for 4 weeks, excluding the trial day. Refer to abbreviations list for the expansion of each neurotransmitter metabolite.

Further analyses of changes in neurotransmitter metabolite concentrations during acute trial days indicated transient, but significant, modulations of select neurotransmitters after BCF consumption. To account for inter-individual variability, metabolite concentrations at 1 h and 2.4 h post dietary intervention consumption were normalized and expressed as percentage changes from acute trial day baseline values. Consistent with results from absolute data, relative 5-HIAA concentrations were significantly lower at 1 h and 2.4 h after BCF supplementation than with the placebo at corresponding timepoints (Figure 7A). Additionally, a significant negative correlation between 5-HIAA and MAO-B inhibition was observed (Figure 7B), further supporting the direct effect of BCF consumption on 5-HT turnover.

**FIGURE 7:**
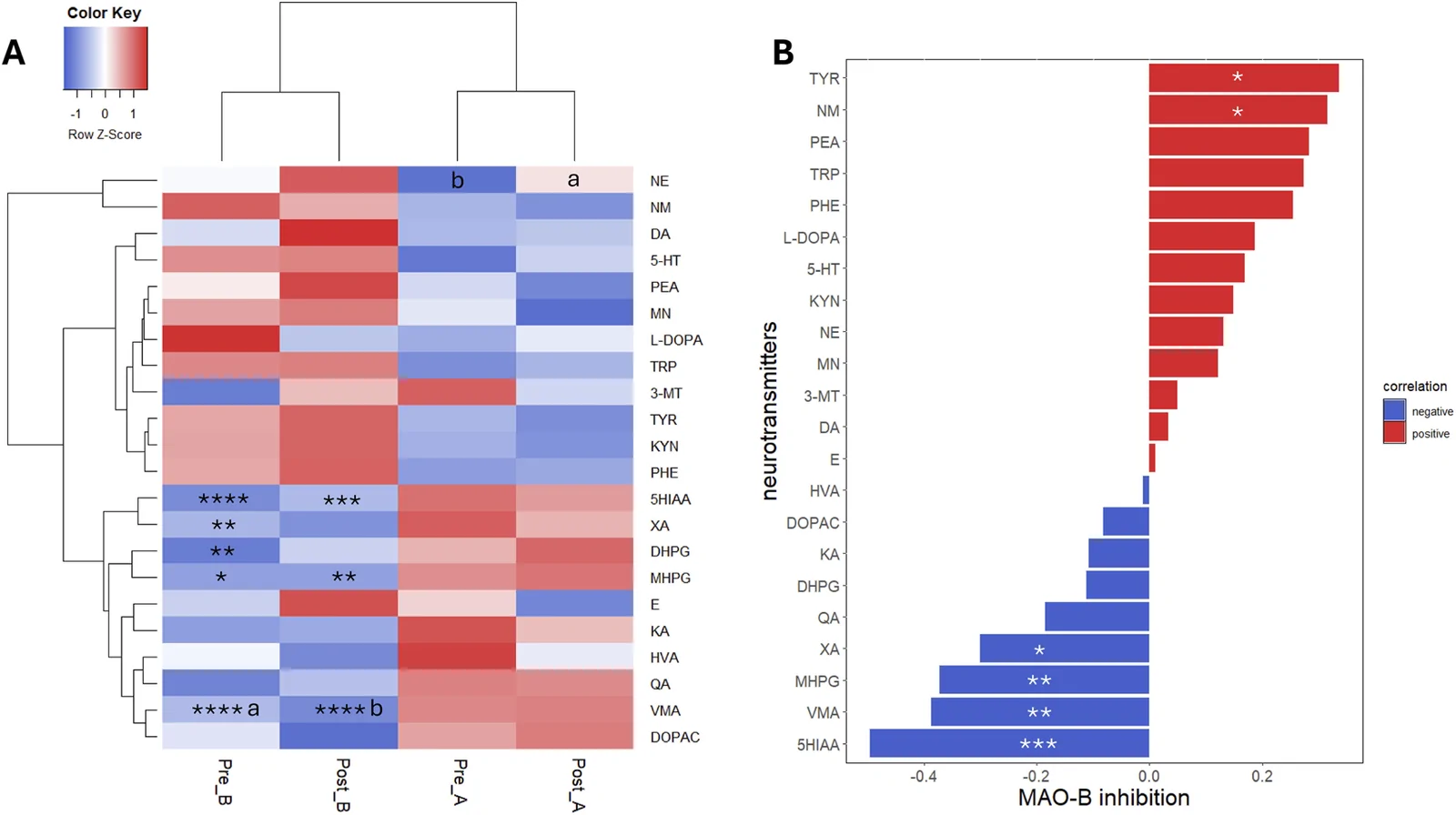
Effects of acute blackcurrant formula (BCF) intervention on circulating neurotransmitters of participants. **(A)** Heatmap visualising relative changes in concentrations from baseline with blues indicating lower and reds higher metabolite scaled Z-score values at Pre and Post cognitive testing; Asterisks indicate statistical significance of pairwise comparisons between paired placebo and BCF samples per time point with *q < 0.05, **q < 0.01, ***q < 0.001, ****q < 0.0001. Letters **(A,B)** indicate statistical significance of pairwise time point comparisons following the consumption of either placebo or BCF, with a symbolising higher and b lower concentrations at α = 0.05. **(B)** Bar graph visualising the strength of Pearsons correlations between each metabolite and MAO-B inhibition, with blues indicating negative and reds positive correlations. Asterisks indicate statistical significance with *q < 0.05, **q < 0.01, ***q < 0.001. Refer to list of abbreviations for the expansion of each neurotransmitter.

The kynurenine (KYN) pathway is the primary route of TRP turnover (Badawy, 2017), and xanthurenic acid (XA), a downstream product of the KYN pathway, showed comparable but shorter-term reductions 1 h after BCF consumption compared with 5-HIAA (Figure 7A). In contrast to 5-HIAA, MAO-A/B enzymes are not involved in the KYN pathway, and the observed significant negative correlations between XA and MAO inhibition (Figure 7B) may be due to confounding factors.

While no significant changes in DA or epinephrine metabolites were observed following acute BCF supplementation, relative DHPG concentrations were significantly lower in the BCF group (5.9% ± 17.4%) than in the placebo group 1 h post supplementation. This was accompanied by significantly lower concentrations of other downstream NE catabolites, including 3-methoxy-4-hydroxyphenylglycol (MHGP) and vanillylmandelic acid (VMA) at 1 h (*p*_adj_ < 0.05 and *p*_adj_ < 0.001, respectively) and 2.4 h (*q* < 0.01 and *q* < 0.001, respectively) following BCF supplementation compared with the placebo (Figure 7A). Both MHPG and VMA were also significantly negatively correlated with MAO-B inhibition (*q* < 0.01; Figure 7B). Collectively, these findings indicate modulation in NE turnover following acute BCF supplementation, which may be facilitated by BCF-induced inhibition of MAO.

### 3.4 Correlations between sleep measures and circulating neurotransmitter profiles

Associations between plasma neurotransmitter concentrations and the primary and secondary outcomes improved by BCF supplementation were examined to determine whether the observed benefits could be explained by diet-induced modulations in circulating neurotransmitters. Sleep quality outcomes from this study cohort were also included in these analyses using data previously reported by Lomiwes et al. (2025b). As these integrative analyses required paired neurotransmitter and outcome data across both intervention periods, only participants with complete data sets were included. Participant data were excluded owing to dietary non-compliance and plasma collection errors, and not based on selective exclusion of data based on participant characteristics or study outcomes. No significant differences were detected between participants included in the integrative analyses and those excluded due to incomplete data with respect to anthropometric characteristics, primary cognitive outcomes, biomarkers of MAO-B inhibition, and selected mood and sleep measures (Supplementary Table S8). These findings indicate that data completeness was independent of participant characteristics and treatment responses, and that participant exclusion due to missing data was unlikely caused attrition bias in the integrative analyses.

Significant associations were measured for sleep quality-related outcomes, which were previously reported to improve following short-term BCF supplementation in this cohort (Lomiwes et al., 2025b). Twelve neurotransmitter metabolites were significantly correlated with total PSQI scores or specific components of sleep quality (Figure 8, Supplementary Table S9). Phenylalanine (PHE), TRP, DA, its precursor L-DOPA, and KYN were positively associated with total PSQI, sleep duration, and subjective sleep quality. Conversely, 5-HT, 5-HIAA, and NE were negatively associated with sleep latency, sleep onset time and habitual sleep efficiency (5-HIAA only). Downstream NE metabolites and MHPG and homovanillic acid (HVA) were also negatively correlated with sleep onset and sleep efficiency, respectively, suggesting a role for NE in specific components of sleep quality.

**FIGURE 8:**
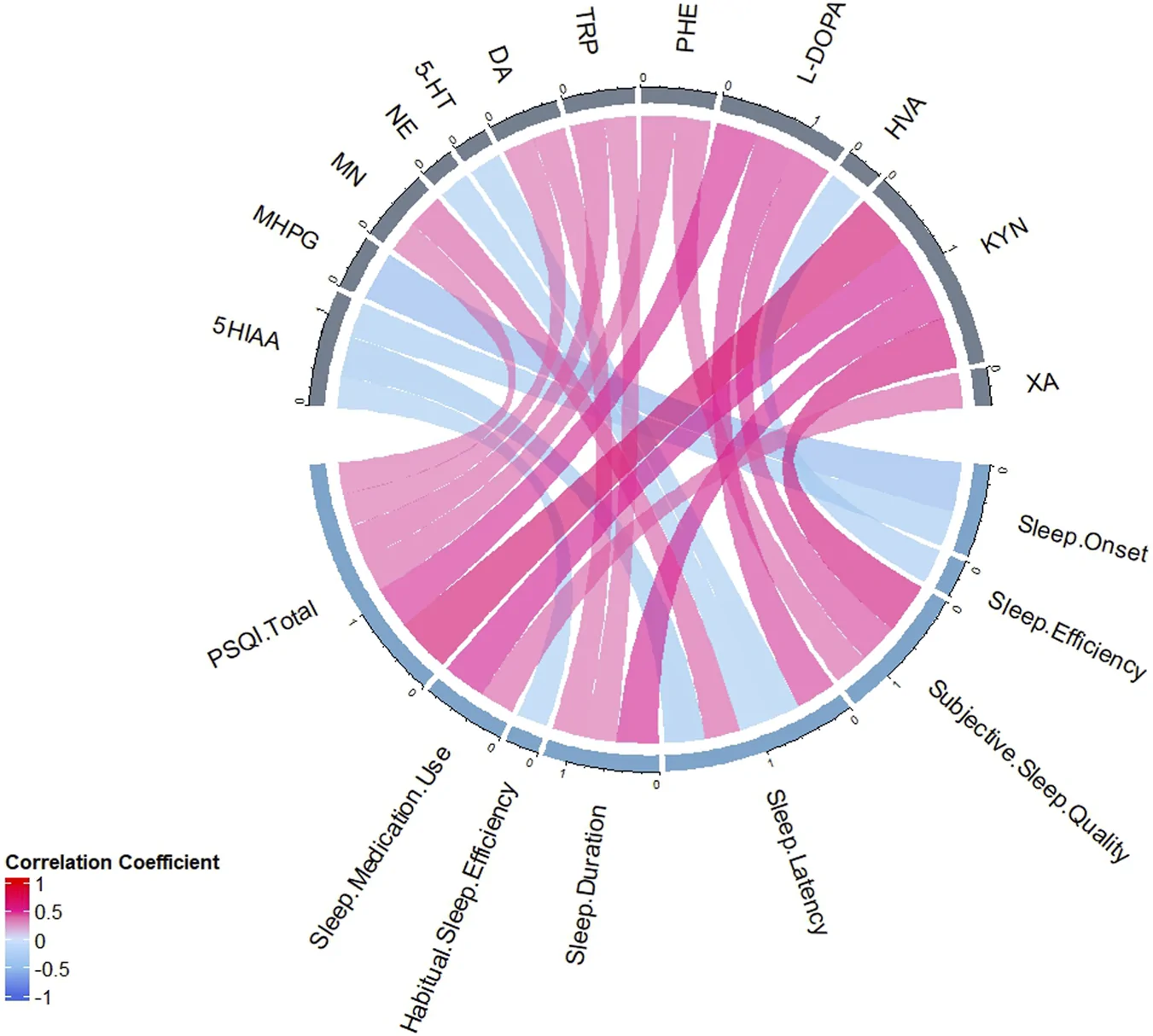
Chord diagram highlighting significant correlations between changes in neurotransmitter concentrations from baseline and sleep quality after 4 weeks of daily consumption of blackcurrant formula or placebo. Warm hues (red) represent positive, and cold hues (blue) negative correlations. The ribbon width reflects the strength of the correlation. Refer to list of abbreviations for the expansion of each neurotransmitter.

## 4 Discussion

To our knowledge this is the first study to demonstrate that four-weeks of BCF supplementation improves attention and vigilance in healthy older adults, alongside a reduction in mental fatigue. These benefits did not correspond with changes in processing speed, indicating that BCF improved cognitive efficiency and resilience, rather than processing speed. Furthermore, accompanying reductions in platelet MAO-B activity and circulating monoamine metabolites support a plausible neurochemical mechanism underlying the observed effects on cognitive performance, mood, and sleep quality.

Although this study was powered to detect changes in Digit Vigilance and RVIP performance, additional cognitive tasks were included to explore the influence of BCF supplementation on cognitive domains beyond attention. Of the cognitive tasks evaluated, RVIP was the only one to show a significant improvement following BCF supplementation. The RVIP task places substantial demands on visual sustained attention and working memory, requiring participants to continuously process information under high cognitive load. Therefore, improvements in RVIP performance provides evidence that BCF supplementation positively influenced attention, the cognitive domain primarily assessed by this task. Importantly, improved RVIP accuracy occurred without a corresponding change in reaction time, indicating enhanced performance was not achieved through a speed/accuracy trade-off.

Improvements in RVIP accuracy was accompanied by a strong trend toward reduced mental fatigue during cognitive testing. Although this effect was not significant following FDR adjustment, the direction of the response suggests that BCF may attenuate mood-related declines during periods of sustained cognitive stress. Together, these findings support the potential application of BCF as a functional food strategy for occupations and everyday activities requiring prolonged attention and mental resilience, such as extended computer-based work, long-duration driving, and shift-based occupations.

Findings from the present study contribute to growing evidence on the potential benefits of polyphenol-rich foods in maintaining mood and cognitive health in older adults (Brito et al., 2023). Randomised controlled studies involving cocoa, cranberries, blueberries and orange juice have reported benefits across attention, working memory and episodic memory domains (Haskell-Ramsay and Docherty, 2023). Similarly, a recent systematic review identified executive function, learning and memory as the cognitive domains most consistently improved by berry supplementation in older adults (Bonyadi et al., 2022). In addition to berry polyphenols, this study utilised a higher anthocyanin dose and included L-theanine and pine bark extract in the active intervention, which may have conferred greater efficacy. L-theanine has previously been associated with improved reaction time during attention-related tasks (Baba et al., 2021), and pine bark extract has been linked to enhanced working memory (Ryan et al., 2008) in older adults.

The only clinical study to date investigating the efficacy of BC in supporting cognitive performance in older adults found no significant improvements in memory and executive function outcomes following 24 weeks supplementation (Do Rosario et al., 2026). The discrepancy between findings reported by Do Rosario et al. (2026) and the present study may be explained by variation in the dose and composition of the active intervention, participant characteristics and study design. Participants in the present study, for example, did not report any memory complaints and were assessed using a different cognitive testing paradigm. These methodological differences highlight the need for consistency in dosing strategies, participant profiles and cognitive assessments across dietary intervention studies to reliably establish cognitive benefits of BCF.

In contrast to younger adult cohorts, where attention-related benefits were observed following an acute dose of BC, improved attention was only observed in the present study following 4 weeks of daily BCF supplementation. This disparity may reflect age-related differences in cognitive function (Jardim et al., 2024) and potential responsiveness to nutritional interventions, suggesting that sustained supplementation may be necessary to achieve measurable improvements in older adults. Differences in task paradigms between studies may also contribute to variability in study findings. Nominal improvements in working and secondary memory observed in this study align with previous evidence demonstrating memory-related benefits of BC and BCF (Gillies et al., 2024; Lomiwes et al., 2025a), respectively. While these effects warrant further investigation, they provide support for the potential of BCF to support memory function, a key cognitive domain vulnerable to age-related decline.

The efficacy of BC and its constituents in supporting cognitive function has been largely credited to its ability to influence neurochemical signalling through inhibition of MAO activity and subsequent modulation of monoamine metabolism (Watson et al., 2015, 2019; 2020; Lomiwes et al., 2019, 2024). Although MAO-inhibitory activity has been reported for a number of edible plant sources *in vitro* (Marzo et al., 2022), such effects have yet to be demonstrated in human dietary intervention studies. In contrast, BC has consistently been shown to inhibit MAO activity *in vivo*, supporting a plausible mechanistic link between BC consumption and improvements in cognitive and mood outcomes. Furthermore, the MAO-B inhibition observed following BCF supplementation is likely driven by BC-derived bioactives, including sarmentosin (Lomiwes et al., 2024), rather than by L-theanine or pine bark extract, which exhibit negligible MAO-B inhibitory bioactivity *in vitro*.

Acute ingestion of BCF was associated with inhibition of platelet MAO-B activity and lower circulating concentrations of the monoamine metabolites 5-HIAA and DHPG, the principal catabolites of 5-HT and NE, respectively (Gaweska and Fitzpatrick, 2011). As both neurotransmitters are preferentially metabolised by MAO-A, peripheral reductions in 5-HIAA and DHPG may serve as indirect indices of MAO-A inhibition and MAO-A-mediated monoamine turnover (Celada et al., 1990; Scheinin et al., 1991; Celada and Artigas, 1993). Although these findings should be interpreted cautiously, the concomitant reduction in platelet MAO-B activity and plasma monoamine metabolites provides further support for previous observations that BC acts as a non-selective MAO inhibitor in humans (Watson et al., 2015; Lomiwes et al., 2024).

The observed reductions in 5-HIAA and DHPG suggest modulation of both serotonergic and noradrenergic tones, which are critically involved in the regulation of cognitive performance, mood, and sleep quality. For example, increased circulating 5-HT from 5-hydroxytrptophan (5-HTP) supplementation modestly improved sleep quality and cognitive performance (Sutanto et al., 2021; Li et al., 2025), and peripheral 5-HT and 5-HIAA have been associated with improved cognitive performance in adults chronically exposed to high altitude conditions (Zhong et al., 2021). Similarly, noradrenergic effects have been associated with several mood and cognition domains, including stress responses (McCall et al., 2015), memory formation (Takeuchi et al., 2016), executive function (Poe et al., 2020) and sleep quality (Aston-Jones and Bloom, 1981). Improvements in cognitive, mood and sleep outcomes from 4 weeks of daily BCF supplementation in this study, may therefore reflect residual MAO-mediated effects from the last BCF dose or potentially modest shifts in basal MAO activity from 4 weeks supplementation.

Improvements in cognitive and mood outcomes following 4 weeks of BCF supplementation may also be mediated by additional mechanisms other than MAO inhibition, consistent with the proposed polyvalent functionality of BC and its constituents. L-theanine has also been demonstrated to increase the metabolism and release of central monoaminergic neurotransmitters (Yokogoshi et al., 1998), likely through mechanisms other than MAO inhibition. Both BC (Lomiwes et al., 2025a) and pine bark extract (Senthilmohan et al., 2003) have strong antioxidant properties that may offer neuro-protective effects from oxidative stress associated aging (Pamu et al., 2025). Furthermore, BC supplementation has been linked to changes in cholinergic metabolism (da Costa et al., 2024), upregulation of BDNF (Morton et al., 2024a), interactions with specific gut microbiota composition (Gillies et al., 2024) and benefits to systemic (Barnes et al., 2020) and central haemodynamics (Watson et al., 2026). Collectively, these findings highlight the polyvalent biological activity of BCF constituents, with their health benefits probably arising from influences on multiple mechanisms involving neurochemical and physiological modulation, antioxidant activity and gut-brain interactions.

While findings from this study are promising, several limitations should be acknowledged. Although the study was adequately powered to detect potential changes in the primary cognitive outcomes, the study cohort was predominately female, which restricts the generalisability of the findings across sexes in this age group (Yom et al., 2022). A more balanced sex distribution may also be particularly important given reported sex-specific differences in cognitive aging, including variations in cognitive strategies (Jockwitz et al., 2021) and rates of decline in domains such as executive function (Levine et al., 2021). Furthermore, while we have previously demonstrated comparable efficacy of acute blackcurrant supplementation on platelet MAO-B activity in males and females (Lomiwes et al., 2024), similar levels of MAO-B inhibition may not necessarily translate to equivalent changes in monoamine metabolism. Reported sex differences in circulating monoamine concentrations (Robinson, 1975) suggest that BCF-mediated MAO inhibition may differentially influence neurotransmitter profiles in males and females, with potential implications for cognitive outcomes that warrant further investigation. Future studies should seek to recruit balanced cohorts or be specifically designed to evaluate potential sex-dependent responses to BCF supplementation.

Although our analyses indicated minimal likelihood of residual carryover between treatment arms following a two-week washout period, the possibility of carryover effects inherent to crossover study designs cannot be completely excluded. Future studies employing a similar study design should consider longer washout periods minimise this potential source of bias. Additionally, longer-term intervention studies are recommended to determine whether the cognitive and neurochemical effects observed from BCF supplementation are maintained or augmented with sustained consumption.

Participant non-compliance, technical issues during blood collection and limited sample volumes substantially reduced the number of participants with complete datasets for neurotransmitter analyses, potentially limiting the broader applicability of these outcomes. Given the considerable inter-individual variability in circulating neurotransmitter concentrations, larger adequately powered studies are needed to definitively characterise the effects of habitual BCF supplementation on circulating neurotransmitter dynamics. Future studies should also incorporate central neuroimaging approaches, such as positron emission tomography (Meyer and Braga, 2021), to better elucidate the relationship between brain MAO activity and peripheral monoamine modulation (Pivac et al., 2006), thereby validating the utility of peripheral monoamines as biomarkers of central monoaminergic tone.

Despite these limitations, the consistency of the cognitive and biological findings provides encouraging evidence that BCF supplementation may support cognitive function in older adults and warrants further investigation in larger, mechanistically focused clinical trials. More broadly, these findings contribute to a growing body of evidence supporting non-pharmacological approaches to sustaining cognitive function during ageing. Given that a substantial proportion of dementia cases are thought to be attributable to modifiable lifestyle, environmental and health-related risk factors (Livingston et al., 2020), there is increasing interest in developing complementary approaches that promote healthy brain aging. Alongside established lifestyle factors such as physical activity, healthy dietary patterns, social engagement, and adequate sleep (Musich et al., 2026), bioactive compounds may offer an additional avenue for preserving cognitive function in later life. Consequently, future research should explore whether integrating BCF supplementation with other lifestyle-based interventions produces additive or synergistic benefits, providing a holistic approach to supporting cognitive resilience and healthy brain ageing in older adults.

## 5 Conclusion

This study demonstrates that 4 weeks of supplementation with a blackcurrant-based formulation (BCF) enriched with L-theanine and pine bark extract confers measurable benefits on cognitive performance, mood and mental resilience in healthy adults aged 55 years and older. These improvements corresponded with transient but significant modulations in peripheral monoamine metabolism and inhibition of platelet MAO-B activity, suggesting that BCF may inhibit both MAO-A and MAO-B isoforms. This dual activity may contribute to the observed improvements in cognitive function, mood and sleep quality in older adults. Collectively, these findings support BCF as a safe and promising nutritional strategy for supporting cognitive health in ageing populations.

## Data Availability

The raw data supporting the conclusions of this article will be made available by the authors, without undue reservation.
