# Supplementary material for: Four-week supplementation with a blackcurrant formulation enhances cognitive performance, reduces mental fatigue and modulates peripheral monoamine metabolism in older adults: a randomized, double-blind, placebo-controlled, crossover study
Source: Front Pharmacol. 2026 Sep 30;17:1896655. doi: 10.3389/fphar.2026.1896655 (PMC13661542; doi:10.3389/fphar.2026.1896655)
Supplement: Supplementary file 1 [file Supplementaryfile1.docx]

Supplementary Material

Table S1. Absolute cognitive outcome scores measured following acute and four‑week daily supplementation with placebo or the blackcurrant formula (BCF).

|  | Placebo | |  | BCF | |  | Time | |  | Treatment | |  | Treatment × Time | |
| --- | --- | --- | --- | --- | --- | --- | --- | --- | --- | --- | --- | --- | --- | --- |
|  | Acute | 4-wk |  | Acute | 4wk |  | *p* value | *q* value |  | *p* value | *q* value |  | *p* value | *q* value |
| Digit vigilance |  |  |  |  |  |  |  |  |  |  |  |  |  |  |
| Correct (%) | 84 ± 3 | 84 ± 3 |  | 82 ± 2 | 82 ± 3 |  | 0.877 | - |  | 0.236 | - |  | 0.959 | - |
| Correct RT (ms) | 530 ± 5 | 534 ± 6 |  | 528 ± 6 | 532 ± 6 |  | 0.180 | - |  | 0.373 | - |  | 0.938 | - |
| False alarms | 4.3 ± 0.7 | 4.3 ± 0.8 |  | 4.8 ± 0.6 | 4.1 ± 0.7 |  | 0.469 | - |  | 0.747 | - |  | 0.519 | - |
|  |  |  |  |  |  |  |  |  |  |  |  |  |  |  |
| RVIP |  |  |  |  |  |  |  |  |  |  |  |  |  |  |
| Correct (%) | 49 ± 5 | 47 ± 5 |  | 48 ± 4 | 51 ± 5 |  | 0.277 | - |  | 0.706 | - |  | 0.185 | - |
| Correct RT (ms) | 593 ± 13 | 584 ± 9 |  | 586 ± 11 | 591 ± 11 |  | 0.703 | - |  | 0.467 | - |  | 0.259 | - |
| False alarms | 5.0 ± 0.9 | 6.0 ± 1 |  | 7.4 ± 1.3 | 5.5 ± 0.9 |  | 0.324 | - |  | 0.128 | - |  | 0.009 | - |
|  |  |  |  |  |  |  |  |  |  |  |  |  |  |  |
| Delayed word recall |  |  |  |  |  |  |  |  |  |  |  |  |  |  |
| Correct | 3.2 ± 0.4 | 3.3 ± 0.4 |  | 3.5 ± 0.4 | 3.9 ± 0.4 |  | 0.367 | 0.626 |  | s | 0.768 |  | 0.519 | 1.00 |
| Error | 1.3 ± 0.4 | 1.5 ± 0.3 |  | 1.2 ± 0.3 | 1.0 ± 0.3 |  | 0.869 | 1.00 |  | 0.19 | 0.936 |  | 0.411 | 1.00 |
|  |  |  |  |  |  |  |  |  |  |  |  |  |  |  |
| Arrow Flankers |  |  |  |  |  |  |  |  |  |  |  |  |  |  |
| Overall accuracy (%) | 100 ± 0.1 | 99 ± 0.2 |  | 100 ± 0.1 | 99 ± 0.2 |  | 0.125 | 0.536 |  | 0.475 | 0.936 |  | 0.598 | 1.00 |
| Overall RT (ms) | 743 ± 30 | 752 ± 28 |  | 744 ± 31 | 741 ± 26 |  | 0.911 | 1.00 |  | 0.627 | 0.936 |  | 0.765 | 1.00 |
| Overall correct RT (ms) | 744 ± 30 | 752 ± 28 |  | 743 ± 31 | 737 ± 25 |  | 0.971 | 1.00 |  | 0.531 | 0.936 |  | 0.680 | 1.00 |
| Congruent – accuracy (%) | 100 ± 0.2 | 100 ± 0 |  | 100 ± 0.2 | 100 ± 0.3 |  | 0.994 | 1.00 |  | 0.166 | 0.936 |  | 0.162 | 1.00 |
| Congruent – overall RT (ms) | 723 ± 35 | 719 ± 22 |  | 742 ± 38 | 704 ± 27 |  | 0.217 | 0.536 |  | 0.962 | 0.972 |  | 0.375 | 1.00 |
| Congruent – correct RT (ms) | 723 ± 35 | 719 ± 22 |  | 742 ± 38 | 704 ± 27 |  | 0.212 | 0.536 |  | 0.941 | 0.972 |  | 0.372 | 1.00 |
| Incongruent – accuracy (%) | 99 ± 0.3 | 99 ± 1 |  | 99 ± 0.3 | 99 ± 1 |  | 0.084 | 0.536 |  | 0.714 | 0.936 |  | 0.872 | 1.00 |
| Incongruent – overall RT (ms) | 785 ± 33 | 811 ± 30 |  | 779 ± 29 | 807 ± 30 |  | 0.194 | 0.536 |  | 0.704 | 0.936 |  | 0.842 | 1.00 |
| Incongruent – correct RT (ms) | 786 ± 33 | 810 ± 29 |  | 780 ± 29 | 798 ± 25 |  | 0.239 | 0.536 |  | 0.559 | 0.936 |  | 0.905 | 1.00 |
| Neutral – accuracy (%) | 100 ± 0.2 | 100 ± 0.2 |  | 100 ± 0.3 | 99 ± 0.3 |  | 0.714 | 1.00 |  | 0.260 | 0.936 |  | 0.691 | 1.00 |
| Neutral – overall RT (ms) | 722 ± 35 | 728 ± 36 |  | 710 ± 29 | 711 ± 29 |  | 0.879 | 1.00 |  | 0.476 | 0.936 |  | 0.947 | 1.00 |
| Neutral – correct RT (ms) | 722 ± 35 | 728 ± 36 |  | 706 ± 29 | 711 ± 28 |  | 0.822 | 1.00 |  | 0.383 | 0.936 |  | 0.991 | 1.00 |
|  |  |  |  |  |  |  |  |  |  |  |  |  |  |  |
| Serial sevens |  |  |  |  |  |  |  |  |  |  |  |  |  |  |
| Total responses | 15 ± 1.3 | 15 ± 1.4 |  | 13 ± 1.0 | 16 ± 1.2 |  | 0.165 | 0.536 |  | 0.850 | 0.954 |  | 0.071 | 0.717 |
| Correct responses | 13 ± 1.3 | 13 ± 1.4 |  | 11 ± 1.0 | 14 ± 1.0 |  | 0.013 | 0.263 |  | 0.403 | 0.936 |  | 0.026 | 0.525 |
| Error responses | 2.3 ± 0.4 | 1.8 ± 0.3 |  | 2.6 ± 0.4 | 2.2 ± 0.7 |  | 0.275 | 0.556 |  | 0.404 | 0.936 |  | 0.933 | 1.00 |
|  |  |  |  |  |  |  |  |  |  |  |  |  |  |  |
| Symbol digit substitution |  |  |  |  |  |  |  |  |  |  |  |  |  |  |
| Time to completion (sec) | 233 ± 9 | 239 ± 11 |  | 236 ± 9 | 241 ± 9 |  | 0.095 | 0.536 |  | 0.772 | 0.936 |  | 0.924 | 1.00 |
| Correct – total (%) | 100 ± 0.1 | 99 ± 0.5 |  | 100 ± 0.1 | 100 ± 0.2 |  | 0.372 | 0.626 |  | 0.788 | 0.936 |  | 0.530 | 1.00 |
| Correct – 90 sec (%) | 99 ± 0.2 | 99 ± 0.5 |  | 100 ± 0.2 | 100 ± 0.2 |  | 0.747 | 1.00 |  | 0.439 | 0.936 |  | 0.997 | 1.00 |

Reaction time, RT. The q values are False Discovery Rate (FDR)-adjusted p values with q < 0.05 indicating significance for main effects effects of treatment and time and their interaction from linear mixed models. Data for each cognitive outcome are means ± SEM.

Table S2. Relative changes in cognitive outcomes following four weeks of daily supplementation with placebo or the blackcurrant formula (BCF), expressed as change from acute trial day scores.

|  | Placebo | BCF | Treatment | |
| --- | --- | --- | --- | --- |
|  |  |  | *p* value | *q* value |
| Δ Digit vigilance |  |  |  |  |
| Correct (%) | −0.34 ± 1.7 | −0.09 ± 2.4 | 0.925 | - |
| Correct RT (ms) | 3.1 ± 3.9 | 3.7 ± 3.3 | 0.904 | - |
| False alarms | 0.04 ± 0.56 | −0.65 ± 0.69 | 0.435 | - |
|  |  |  |  |  |
| Δ RVIP |  |  |  |  |
| Correct (%) | −1.7 ± 2.4 | 0.8 ± 3.4 | 0.549 | - |
| Correct RT (ms) | −14 ± 9 | −18 ± 24 | 0.825 | - |
| False alarms | 0.7 ± 0.7 | −2.3 ± 0.7 | 0.007 | - |
|  |  |  |  |  |
| Δ Delayed word recall |  |  |  |  |
| Correct | 0.06 ± 0.27 | 0.35 ± 0.25 | 0.436 | 0.953 |
| Error | 0.23 ± 0.23 | −0.15 ± 0.38 | 0.390 | 0.953 |
|  |  |  |  |  |
| Δ Arrow Flankers |  |  |  |  |
| Overall accuracy (%) | −0.2 ± 0.22 | −0.3 ± 0.21 | 0.694 | 1.00 |
| Overall RT (ms) | 6.2 ± 25 | −2.8 ± 21 | 0.668 | 1.00 |
| Overall correct RT (ms) | 5.4 ± 24 | −5.3 ± 19 | 0.647 | 1.00 |
| Congruent – accuracy (%) | 0.2 ± 0.2 | −0.2 ± 0.2 | 0.161 | 0.953 |
| Congruent – overall RT (ms) | −8.3 ± 28 | −37.9 ± 25 | 0.335 | 0.953 |
| Congruent – correct RT (ms) | −8.4 ± 28 | −38 ± 25 | 0.332 | 0.953 |
| Incongruent – accuracy (%) | −0.8 ± 0.6 | −0.6 ± 0.5 | 0.767 | 1.00 |
| Incongruent – overall RT (ms) | 24 ± 28 | 28 ± 28 | 0.971 | 1.00 |
| Incongruent – correct RT (ms) | 22 ± 27 | 18 ± 23 | 0.879 | 1.00 |
| Neutral – accuracy (%) | 0 ± 0.3 | −0.2 ± 0.4 | 0.72 | 1.00 |
| Neutral – overall RT (ms) | 3.3 ± 37 | 1.8 ± 24 | 0.972 | 1.00 |
| Neutral – correct RT (ms) | 3.4 ± 37 | 4.8 ± 23 | 0.948 | 1.00 |
|  |  |  |  |  |
| Δ Serial sevens |  |  |  |  |
| Total responses | −0.2 ± 1 | 2.4 ± 1.1 | 0.076 | 0.800 |
| Correct responses | 0.3 ± 0.9 | 2.8 ± 0.7 | 0.025 | 0.511 |
| Error responses | −0.5 ± 0.5 | −0.4 ± 0.6 | 0.904 | 1.00 |
|  |  |  |  |  |
| Δ Symbol digit substitution |  |  |  |  |
| Time to completion (sec) | −1.4 ± 1.1 | −0.1 ± 0.2 | 0.876 | 1.00 |
| Correct – total (%) | −1.3 ± 1 | −0.1 ± 0.2 | 0.245 | 0.953 |
| Correct – 90 sec (%) | −1 ± 1.1 | −0.1 ± 0.2 | 0.399 | 0.953 |

Reaction time, RT; Rapid Visual Information Processing, RVIP. The q values are False Discovery Rate (FDR)-adjusted p-values for main effects of treatment from linear mixed models with q < 0.05 indicating significant treatment effect. Data are means ± SEM.

Table S3. Bond–Lader composite mood scores and individual Visual Analogue Mood Scales (VAMS) scores measured pre‑ and post‑cognitive testing on trial days following acute and four‑week supplementation with placebo or the blackcurrant formula (BCF).

|  | Placebo | | | |  | BCF | | | | Time | Treatment | Repetition | Timepoint × Treatment | Timepoint × Treatment × Repetition |
| --- | --- | --- | --- | --- | --- | --- | --- | --- | --- | --- | --- | --- | --- | --- |
|  | Acute | | 4 wk | |  | Acute | | 4 wk | |  |  |  |  |  |
|  | Pre | Post | Pre | Post |  | Pre | Post | Pre | Post |  |  |  |  |  |
| Bond Lader (composite score) |  |  |  |  |  |  |  |  |  |  |  |  |  |  |
| Alertness | 73 ± 3 | 68 ± 3 | 77 ± 3 | 72 ± 3 |  | 72 ± 4 | 67 ± 4 | 74 ± 3 | 71 ± 3 | 0.008  (0.048) | 0.162  (0.245) | 0.001  (0.001) | 0.911  (0.920) | 0.818  (0.885) |
| Stress | 28 ± 3 | 33 ± 4 | 24 ± 4 | 32 ± 4 |  | 28 ± 4 | 37 ± 4 | 27 ± 3 | 31 ± 4 | 0.054  (0.162) | 0.348  (0.351) | <0.001  (<0.001) | 0.696  (0.920) | 0.152  (0.459) |
| Contentment | 81 ± 2 | 76 ± 3 | 84 ± 2 | 79 ± 3 |  | 82 ± 3 | 75 ± 3 | 81 ± 2 | 77 ± 3 | 0.111  (0.225) | 0.344  (0.351) | <0.001  (<0.001) | 0.361  (0.920) | 0.393  (0.793) |
|  |  |  |  |  |  |  |  |  |  |  |  |  |  |  |
| VAMS (mm) |  |  |  |  |  |  |  |  |  |  |  |  |  |  |
| Stress | 19 ± 3 | 28 ± 4 | 22 ± 4 | 24 ± 4 |  | 23 ± 5 | 32 ± 4 | 24 ± 3 | 27 ± 4 | 0.552  (0.558) | 0.114  (0.245) | 0.007  (0.007) | 0.546  (0.920) | 0.876  (0.885) |
| Anxious | 21 ± 3 | 27 ± 4 | 17 ± 3 | 26 ± 4 |  | 24 ± 4 | 29 ± 3 | 22 ± 4 | 27 ± 4 | 0.253  (0.383) | 0.136  (0.245) | <0.001  (<0.001) | 0.900  (0.920) | 0.649  (0.885) |
| Mentally fatigued | 24 ± 3 | 32 ± 4 | 22 ± 4 | 33 ± 4 |  | 26 ± 5 | 39 ± 5 | 27 ± 4 | 31 ± 4 | 0.350  (0.424) | 0.086  (0.245) | <0.001  (<0.001) | 0.373  (0.920) | 0.086  (0.459) |

The p values for main effects of treatment, time and repetition and their interaction from linear mixed models are presented and the *q* values (in parentheses) are False Discovery Rate (FDR)-adjusted p-values. Data for each mood outcome are means ± SEM.

Table S4. Relative changes in Bond–Lader composite scores and individual Visual Analogue Mood Scales (VAMS), expressed as change from pre‑cognitive test baseline, on trial days following acute and four‑week supplementation with placebo or the blackcurrant formula (BCF). Significance (*p* values) for main effects of treatment, time and repetition and their interaction from linear mixed models are also presented. Data for mood outcomes are means ± SEM.

|  | Placebo | |  | BCF | |  | Time | |  | Treatment | |  | Time × Treatment | |
| --- | --- | --- | --- | --- | --- | --- | --- | --- | --- | --- | --- | --- | --- | --- |
|  | Acute | 4 wk |  | Acute | 4 wk |  | p value | q value |  | p value | q value |  | p value | q value |
| Δ Bond Lader (composite score) |  |  |  |  |  |  |  |  |  |  |  |  |  |  |
| Alertness | −5 ± 1.7 | −4.8 ± 1.2 |  | −5.2 ± 2.3 | −3.8 ± 1.8 |  | 0.564 | 0.684 |  | 0.784 | 0.924 |  | 0.664 | 0.805 |
| Stress | 5.5 ± 1.7 | 8.4 ± 1.9 |  | 9.5 ± 2.9 | 3.6 ± 2.1 |  | 0.473 | 0.684 |  | 0.855 | 0.924 |  | 0.042 | 0.127 |
| Contentment | −4.6 ± 1.8 | −4.9 ± 1.2 |  | −7.5 ± 2 | −4.3 ± 1.3 |  | 0.253 | 0.511 |  | 0.352 | 0.924 |  | 0.167 | 0.337 |
|  |  |  |  |  |  |  |  |  |  |  |  |  |  |  |
| Δ VAMS (mm) |  |  |  |  |  |  |  |  |  |  |  |  |  |  |
| Stress | 8.7 ± 3.1 | 2.1 ± 4.5 |  | 8.7 ± 3.9 | 3.4 ± 3.5 |  | 0.108 | 0.511 |  | 0.85 | 0.924 |  | 0.858 | 0.867 |
| Anxious | 6.3 ± 2.9 | 9.0 ± 3.0 |  | 5.8 ± 2.7 | 5.2 ± 2.7 |  | 0.703 | 0.710 |  | 0.442 | 0.924 |  | 0.565 | 0.805 |
| Mentally fatigued | 7.8 ± 2.7 | 11.1 ± 2.7 |  | 13.6 ± 3 | 4.8 ± 1.9 |  | 0.243 | 0.511 |  | 0.915 | 0.924 |  | 0.011 | 0.067 |

The q values are False Discovery Rate (FDR)-adjusted p-values for main effects of time, treatment, and their interaction from linear mixed models with q < 0.05 indicating significant treatment effect. Data are means ± SEM.

Table S5: Carryover effects analyses of cognitive, mood, and biomarkers of MAO inhibition over the course of this study.

|  | Order | |  | Order × Trial Day | |
| --- | --- | --- | --- | --- | --- |
|  | *p* value | *q* value |  | *p* value | *q* value |
| Digit vigilance |  |  |  |  |  |
| Correct (%) | 0.640 | 0.807 |  | 0.412 | 0.861 |
| Correct RT (ms) | 0.528 | 0.789 |  | 0.471 | 0.861 |
| False alarms | 0.412 | 0.786 |  | 0.325 | 0.861 |
|  |  |  |  |  |  |
| RVIP |  |  |  |  |  |
| Correct (%) | 0.093 | 0.656 |  | 0.852 | 0.995 |
| Correct RT (ms) | 0.648 | 0.807 |  | 0.466 | 0.861 |
| False alarms | 0.698 | 0.841 |  | 0.114 | 0.861 |
|  |  |  |  |  |  |
| Delayed word recall |  |  |  |  |  |
| Correct | 0.563 | 0.799 |  | 0.222 | 0.861 |
| Error | 0.257 | 0.786 |  | 0.618 | 0.930 |
|  |  |  |  |  |  |
| Arrow Flankers |  |  |  |  |  |
| Overall accuracy (%) | 0.065 | 0.656 |  | 0.766 | 0.987 |
| Overall RT (ms) | 0.158 | 0.656 |  | 0.812 | 0.993 |
| Overall correct RT (ms) | 0.154 | 0.656 |  | 0.737 | 0.984 |
| Congruent – accuracy (%) | 0.208 | 0.727 |  | 1.000 | 1.000 |
| Congruent – overall RT (ms) | 0.103 | 0.656 |  | 0.346 | 0.861 |
| Congruent – correct RT (ms) | 0.102 | 0.656 |  | 0.335 | 0.861 |
| Incongruent – accuracy (%) | 0.008 | 0.299 |  | 0.901 | 1.000 |
| Incongruent – overall RT (ms) | 0.134 | 0.656 |  | 0.970 | 1.000 |
| Incongruent – correct RT (ms) | 0.142 | 0.656 |  | 0.969 | 1.000 |
| Neutral – accuracy (%) | 0.484 | 0.786 |  | 0.484 | 0.861 |
| Neutral – overall RT (ms) | 0.386 | 0.786 |  | 0.702 | 0.984 |
| Neutral – correct RT (ms) | 0.355 | 0.786 |  | 0.482 | 0.861 |
|  |  |  |  |  |  |
| Serial sevens |  |  |  |  |  |
| Total responses | 0.433 | 0.786 |  | 0.103 | 0.861 |
| Correct responses | 0.476 | 0.786 |  | 0.068 | 0.861 |
| Error responses | 0.799 | 0.905 |  | 0.731 | 0.984 |
|  |  |  |  |  |  |
| Symbol digit substitution |  |  |  |  |  |
| Time to completion (sec) | 0.980 | 1.000 |  | 0.622 | 0.930 |
| Correct – total (%) | 0.457 | 0.786 |  | 0.446 | 0.861 |
| Correct – 90 sec (%) | 0.627 | 0.807 |  | 0.423 | 0.861 |
|  |  |  |  |  |  |
| Δ Bond-Lader |  |  |  |  |  |
| Alertness | 0.799 | 0.905 |  | 0.610 | 0.930 |
| Stress | 0.894 | 0.955 |  | 0.361 | 0.861 |
| Contentment | 0.467 | 0.786 |  | 0.341 | 0.861 |
|  |  |  |  |  |  |
| Δ VAMS |  |  |  |  |  |
| Stress | 0.279 | 0.786 |  | 0.294 | 0.861 |
| Anxious | 0.577 | 0.799 |  | 0.921 | 1.000 |
| Mentally fatigued | 0.214 | 0.727 |  | 0.220 | 0.861 |
|  |  |  |  |  |  |
| MAO-B activity | 0.352 | 0.786 |  | 0.824 | 0.993 |
|  |  |  |  |  |  |
| Neurotransmitters |  |  |  |  |  |
| 5-HIAA (mmol/L) | 0.519 | 0.789 |  | 0.085 | 0.861 |
| DHPG (mmol/L) | 0.869 | 0.955 |  | 0.436 | 0.861 |

Reaction time, RT; Rapid Visual Information Processing, RVIP. The q values are False Discovery Rate (FDR)-adjusted p-values for main effects of treatment Order and Order × Trial Day interaction from linear mixed models with q < 0.05 indicating significant treatment effect.

Table S6. Concentrations of total anthocyanins, sarmentosin and L-theanine in plasma collected at allocated time points following acute supplementation (baseline [BL], 1 h, and 2.4 h) and after four weeks of supplementation (BL) with either placebo or the blackcurrant formula (BCF). Significance for main effects of time for the BCF treatment only is also presented. Data for bioavailability outcomes are means ± SEM.

|  | Placebo | | | |  | BCF | | | |  | Effect of Time  (for BCF treatment only) | |
| --- | --- | --- | --- | --- | --- | --- | --- | --- | --- | --- | --- | --- |
|  | Acute | | | 4 wk |  | Acute | | | 4 wk |  |  |  |
|  | BL | 1 h | 2.4 h | BL |  | BL | 1 h | 2.4 h | BL |  | p value | q value |
| Anthocyanin  (nM Cy-glu equivalents) | nd | nd | 0.06 ± <0.01 | nd |  | 0.03 ± 0.01 | 16 ± 6 | 9.7 ± 1.1 | 1 ± 0.7 |  | 0.001 | <0.001 |
| Sarmentosin (nM) | nd | nd | nd | nd |  | 0 ± 0 | 442 ± 41 | 323 ± 30 | 29 ± 23 |  | <0.001 | <0.001 |
| L-theanine (µM) | nd | nd | nd | nd |  | 0.1 ± 0.1 | 32 ± 1 | 23 ± 1 | 2.7 ± 1.9 |  | <0.001 | <0.001 |

Table S7. Absolute platelet monoamine oxidase (MAO)-B activity and plasma concentrations of 5-hydroxyindoleacetic acid (5-HIAA) and reducing 3,5-dihydroxyphenylglycine (DHPG) at allocated timepoints following acute supplementation (baseline [BL], 1 h, and 2.4 h) and after four weeks of supplementation (BL) with either placebo or the blackcurrant formula (BCF). Significance (*p* values) for main effects of treatment and time and their interaction from linear mixed models are also presented. Data for biomarkers of MAO-B and MAO-A inhibition are means ± SEM.

|  | Placebo | | | |  | BCF | | | | Time | Treatment | Treatment × Time |
| --- | --- | --- | --- | --- | --- | --- | --- | --- | --- | --- | --- | --- |
|  | Acute | | | 4 wk |  | Acute | | | 4 wk |  |  |  |
|  | BL | 1 h | 2.4 h | BL |  | BL | 1 h | 2.4 h | BL |  |  |  |
| MAO B activity | 19.2 ± 1.4 | 19.3 ± 1.3 | 17.8 ± 1.6 | 20.3 ± 1.4 |  | 19.3 ± 1.4 | 1.5 ± 0.2 | 3.4 ± 0.5 | 16.3 ± 1.3 | <0.001  (<0.001) | <0.001  (<0.001) | <0.001  (<0.001) |
| 5-HIAA (mmol/L) | 27 ± 2 | 28 ± 2 | 26 ± 2 | 26 ± 2 |  | 31 ± 2 | 20 ± 2 | 22 ± 2 | 28 ± 2 | <0.001  (<0.001) | 0.043  (0.014) | <0.001  (<0.001) |
| DHPG (mmol/L) | 5.8 ± 0.5 | 5.8 ± 0.4 | 6.2 ± 0.5 | 5.2 ± 0.5 |  | 5.3 ± 0.3 | 4.5 ± 0.4 | 5 ± 0.5 | 5.6 ± 0.4 | 0.345  (0.116) | 0.001  (<0.001) | 0.008  (0.008) |

Table S8. Key anthropometric, cognitive, mood, and sleep quality parameters of participants included in and excluded from the integrated paired-data analysis. Except for anthropometric measures, which were assessed at baseline, values are mean from data collected on both the acute and four-week trial days across the placebo and blackcurrant formula (BCF) treatment arms.

|  | Integrated paired data analyses | |  | p value |
| --- | --- | --- | --- | --- |
|  | Included (n = 18) | Excluded (n = 6) |  |  |
| Anthropometric measures |  |  |  |  |
| Sex (male/female) | 7/11 | 1/7 |  | - |
| Age (years) | 62 ± 1.4 | 63 ± 2.2 |  | 0.842 |
| BMI (kg/m^2^) | 27.8 ± 3.6 | 30.0 ± 2.9 |  | 0.394 |
|  |  |  |  |  |
| Δ RVIP |  |  |  |  |
| Correct (%) | -1.3 ± 2.5 | 1.5 ± 3.5 |  | 0.536 |
| Correct RT (ms) | -20.7 ± 17.0 | -6.0 ± 15.9 |  | 0.629 |
| False alarms | -0.70 ± 0.70 | -1.1 ± 0.80 |  | 0.695 |
|  |  |  |  |  |
| Δ Digit Vigilance |  |  |  |  |
| Correct (%) | -1.1 ± 1.6 | 1.8 ± 3.0 |  | 0.410 |
| Correct RT (ms) | 0.70 ± 2.8 | 9.6 ± 4.9 |  | 0.103 |
| False alarms | -0.25 ± 0.51 | -0.44 ± 0.90 |  | 0.853 |
|  |  |  |  |  |
| Δ Bond Lader |  |  |  |  |
| Stress | 7.0 ± 1.3 | 6.2 ± 2.2 |  | 0.772 |
|  |  |  |  |  |
| Δ VAMS |  |  |  |  |
| Mental Fatigue | 8.6 ± 1.5 | 10.8 ± 2.7 |  | 0.563 |
|  |  |  |  |  |
| PSQI |  |  |  |  |
| Total PSQI score | 11.5 ± 0.5 | 11.7 ± 0.5 |  | 0.839 |
| Habitual sleep efficiency | 1.2 ± 0.1 | 0.90 ± 0.22 |  | 0.505 |

Body mass index, BMI; reaction time, RT; Visual Analogue Mood Scales, VAMS; Pittsburgh Sleep Quality Index, PSQI. Outcomes with a p < 0.05 indicate significant difference between data sets included in and excluded from integrated paired data analyses. Data are means ± SEM.

Table S9. Pearson correlations between changes from baseline in neurotransmitter concentrations and sleep quality after 4 weeks of daily supplementation with either the blackcurrant formulation or placebo. Values are presented as correlation coefficients (*r*), with corresponding *q*-values shown in parentheses.

|  | 5-HIAA | MHPG | MN | NE | 5-HT | DA | TRP | PHE | L-DOPA | HVA | KYN | XA |
| --- | --- | --- | --- | --- | --- | --- | --- | --- | --- | --- | --- | --- |
| Sleep onset | 0.42 | 0.58 | -0.14 | 0.25 | -0.01 | -0.2 | -0.04 | -0.09 | -0.26 | 0.07 | -0.24 | -0.11 |
|  | (0.022) | (0.001) | (0.463) | (0.187) | (0.942) | (0.302) | (0.832) | (0.620) | (0.167) | (0.703) | (0.194) | (0.555) |
| Sleep duration | -0.16 | -0.17 | 0.18 | 0.19 | 0.01 | 0.19 | 0.33 | 0.09 | 0.24 | -0.27 | 0.36 | 0.01 |
|  | (0.386) | (0.377) | (0.350) | (0.322) | (0.947) | (0.319) | (0.079) | (0.634) | (0.205) | (0.157) | (0.054) | (0.963) |
| Habitual sleep efficiency | -0.32 | -0.23 | 0.06 | -0.04 | 0.02 | -0.01 | 0.18 | 0.13 | 0.16 | -0.4 | 0.24 | -0.24 |
|  | (0.087) | (0.226) | (0.770) | (0.832) | (0.929) | (0.942) | (0.333) | (0.493) | (0.401) | (0.028) | (0.192) | (0.192) |
| Subjective sleep quality | -0.04 | -0.09 | -0.18 | -0.16 | 0.27 | -0.31 | -0.35 | -0.41 | -0.46 | -0.14 | -0.6 | -0.26 |
|  | (0.827) | (0.633) | (0.353) | (0.401) | (0.155) | (0.095) | (0.058) | (0.025) | (0.010) | (0.473) | (0.001) | (0.172) |
| Sleep latency | 0.47 | 0.26 | -0.41 | 0.36 | 0.38 | -0.32 | -0.2 | -0.25 | -0.47 | 0.08 | -0.26 | 0.19 |
|  | (0.008) | (0.167) | (0.023) | (0.049) | (0.041) | (0.086) | (0.297) | (0.176) | (0.009) | (0.658) | (0.164) | (0.311) |
| Sleep duration | 0.22 | 0.27 | -0.32 | -0.17 | 0.04 | -0.38 | -0.38 | -0.2 | -0.35 | 0.18 | -0.5 | -0.03 |
|  | (0.248) | (0.144) | (0.085) | (0.373) | (0.831) | (0.037) | (0.040) | (0.293) | (0.058) | (0.346) | (0.005) | (0.859) |
| Sleep efficiency | 0.37 | 0.21 | -0.20 | 0.06 | 0.04 | -0.11 | -0.31 | -0.23 | -0.32 | 0.23 | -0.31 | 0.36 |
|  | (0.047) | (0.261) | (0.287) | (0.742) | (0.818) | (0.558) | (0.097) | (0.221) | (0.087) | (0.224) | (0.092) | (0.054) |
| Sleep disturbances | 0.13 | 0.07 | -0.16 | 0.28 | 0.11 | -0.15 | 0.06 | -0.27 | -0.02 | -0.31 | -0.22 | 0.2 |
|  | (0.492) | (0.729) | (0.386) | (0.130) | (0.567) | (0.436) | (0.738) | (0.148) | (0.914) | (0.099) | (0.248) | (0.290) |
| Sleep medication use | 0.23 | 0.25 | -0.17 | 0.08 | 0.01 | -0.19 | -0.12 | -0.18 | -0.25 | 0.08 | -0.5 | -0.41 |
|  | (0.218) | (0.187) | (0.362) | (0.667) | (0.974) | (0.316) | (0.543) | (0.341) | (0.190) | (0.683) | (0.005) | (0.024) |
| Daytime function | -0.24 | -0.19 | 0.06 | -0.24 | -0.29 | -0.01 | -0.22 | -0.10 | -0.003 | -0.23 | -0.15 | 0.004 |
|  | (0.196) | (0.306) | (0.746) | (0.205) | (0.124) | (0.937) | (0.236) | (0.583) | (0.986) | (0.222) | (0.432) | (0.982) |
| PSQI total score | 0.35 | 0.25 | -0.37 | 0.06 | 0.13 | -0.38 | -0.40 | -0.40 | -0.49 | 0.05 | -0.65 | 0.06 |
|  | (0.056) | (0.176) | (0.042) | (0.734) | (0.501) | (0.041) | (0.027) | (0.027) | (0.006) | (0.799) | (<0.001) | (0.766) |

5-hydroxyindoleacetic acid, 5-HIAA; 3-methoxy-4-hydroxyphenylethylene glycol, MHPG; metanephrine, MN; norepinephrine, NE; serotonin, 5-HT; dopamine, DA; tryptophan, TRP; phenylalanine, PHE; 3,4-dihydroxyphenylalanine, L-DOPA; homovanillic acid, HVA; kynurenine, KYN; xanthurenic acid, XA; Pittsburgh Sleep Quality Index, PSQI
